# Targeting MDSCs in cancer: emerging immunotherapeutic and metabolic strategies

**DOI:** 10.3389/fimmu.2026.1749965

**Published:** 2026-02-27

**Authors:** Shubhankar Dash, Patryk Firmanty, Monika Chomczyk, Vakul Mohanty, Wenxue Ma, Natalia Baran

**Affiliations:** 1 Department of Experimental Hematology, Institute of Hematology and Transfusion Medicine, Warsaw, Poland; 2 Translational Medicine Doctoral School, Centre of Postgraduate Medical Education, Warsaw, Poland; 3 Department of Bioinformatics and Computational Biology, The University of Texas MD Anderson Cancer Center, Houston, TX, United States; 4 Sanford Stem Cell Institute, Department of Medicine and Moores Cancer Center, University of California, San Diego, San Diego, CA, United States; 5 Department of Hematology and Central Hematology Laboratory, InselSpital, Bern University Hospital, University of Bern, Bern, Switzerland; 6 Department of Internal Medicine, Sanford School of Medicine, The University of South Dakota, Sioux Falls, SD, United States

**Keywords:** immune checkpoint blockade, immunometabolism, cancer immunotherapy, metabolic reprogramming, myeloid-derived suppressor cells (MDSCs), tumor microenvironment (TME), tumor immune evasion, biomarker-driven therapy

## Abstract

Myeloid-derived suppressor cells (MDSCs) are a diverse group of immature myeloid cells critically involved in establishing an immunosuppressive environment within tumors. They impede effective anti-tumor immune responses through multiple mechanisms, including metabolic reprogramming, cytokine secretion, and immune checkpoint ligand expression. This immunosuppressive activity enables tumor progression and resistance to therapies, including immunotherapy. Recent advances reveal that targeting the metabolic pathways of MDSCs can impair their suppressive functions, offering promising strategies to enhance anti-cancer immunity. Approaches such as metabolic inhibition, direct depletion, blockade of recruitment and expansion, and promotion of differentiation into mature immune cells are under active investigation. Combining these strategies with immune checkpoint inhibitors and cell-based therapies, such as cancer vaccines and adoptive T-cell or NK-cell therapies, holds significant potential for overcoming immune resistance. Nonetheless, challenges including MDSC heterogeneity, toxicity, and biomarker validation must be addressed to optimize clinical translation. This review comprehensively covers current insights into the immune-metabolic mechanisms underpinning MDSC-mediated immunosuppression in the tumor microenvironment. It explores emerging therapeutic strategies aimed at targeting MDSCs through metabolic interventions, depletion, and modulation of their recruitment and differentiation. Furthermore, it discusses the integration of MDSC-targeted approaches with existing immunotherapies, highlights ongoing clinical trials, and assesses future directions, such as personalized, biomarker-driven treatments. Ultimately, this review underscores the potential of MDSC-focused therapies to significantly improve the efficacy of cancer immunotherapy and overcome mechanisms of tumor immune evasion.

## Introduction

The tumor microenvironment (TME) is a dynamic, heterogeneous ecosystem in which malignant cells coexist with stromal elements, vasculature, and a spectrum of immune cell populations. It encompasses immune cell subsets with immunostimulatory and immunosuppressive activities (1, 2). In many solid tumors, the TME evolves into an immunosuppressive niche, characterized by nutrient depletion, hypoxia, suppressive cytokines, and the accumulation of immunosuppressive cells, which allow cancer to evade anti-tumor immune responses and consequently limit the efficacy of anticancer therapies (1). Myeloid-derived suppressor cells (MDSCs) are a heterogeneous population of immature myeloid-derived immune cells that play a prominent role in driving immune suppression and limiting the response to immunotherapies (3, 4). These heterogeneous, pathologically activated immature myeloid cells (monocytic and polymorphonuclear subsets) employ multiple mechanisms – arginine depletion, reactive oxygen and nitrogen species, immune-checkpoint ligand expression, and secretion of suppressive cytokines and lipid mediators – to inhibit T cell activation and support tumor progression. MDSC abundance and activity are correlated with poor prognosis and a reduced response to immune checkpoint blockade across several cancer types (5).

Metabolic reprogramming is a key axis by which the TME enforces MDSC function. Rather than merely passive bystanders, MDSCs rewire their metabolism to survive in a nutrient-depleted TME (6). The rewired metabolic adaptation also enables MDSCs to generate immunosuppressive mediators (e.g., PGE2, arginase 1 (ARG1), NOS), which together support MDSC persistence in hostile microenvironments and directly impair effector lymphocyte metabolism and function (6). Since MDSCs are at the intersection of cellular immunosuppression and metabolic dysregulation, they represent an attractive therapeutic target to broaden and deepen anti-tumor immunity (7). Preclinical and early clinical strategies that deplete MDSCs, block their recruitment, inhibit key metabolic pathways, or reprogram them toward differentiation have shown promise in restoring T-cell activity and improving responses to checkpoint inhibitors and other therapies (8). Nevertheless, clinical translation faces considerable challenges, including the heterogeneity of MDSCs, overlapping expression of metabolic targets across cell types, and potential systemic toxicities that limit clinical therapeutic outcomes. Therefore, rational combination approaches that concurrently target MDSCs and the complex immunosuppressive network in the TME, together with biomarker-guided patient selection to enable personalized therapies, are critical for clinical success (7).

In this review, we explore recent insights into the immunometabolic characteristics of MDSCs, focusing on newly identified features in metabolism and immune regulation. We also discuss therapeutic strategies targeting metabolic and immune pathways, including metabolic targeting of MDSCs, strategies to deplete MDSC populations, blocking their recruitment into the TME, and promoting their differentiation into mature non-immunosuppressive myeloid cells. We highlight novel approaches that combine immune checkpoint blockade and chimeric antigen receptor (CAR)-based therapies with MDSC-targeted approaches. We also discuss ongoing clinical trials that target MDSCs immune and metabolic regulation, with the aim of improving the efficacy and clinical outcomes of immunotherapies in hematological and solid malignancies. Finally, we address future perspectives and challenges associated with the immunometabolic targeting of MDSCs in cancer treatment.

## Ontogeny and classification of MDSCs

**Figure 1:**
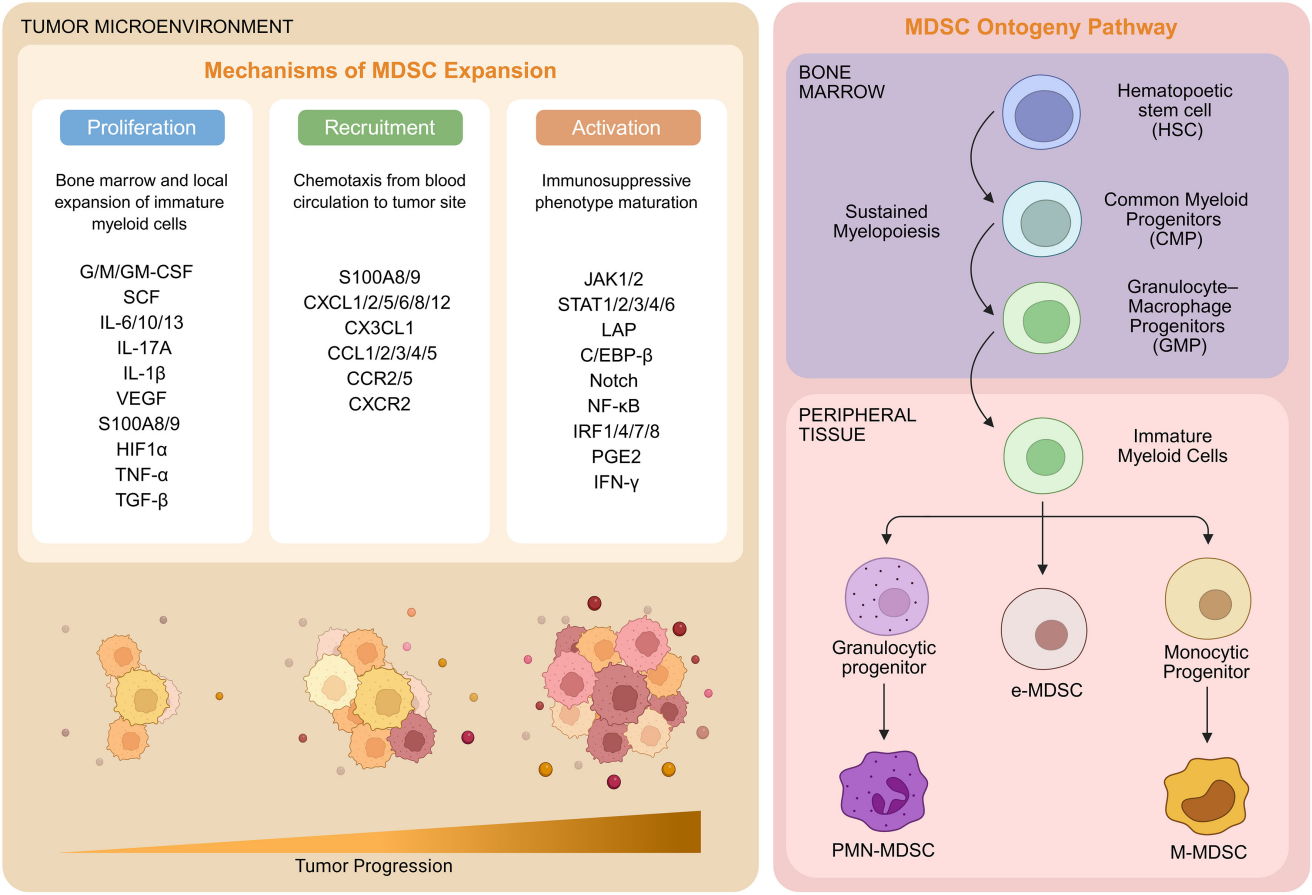
Ontogeny and expansion mechanisms of MDSCs in the tumor microenvironment. (Left panel) Under chronic pathological conditions, expansion of MDSCs occurs through three parallel and simultaneous mechanisms: proliferation (blue), recruitment (green), and activation (orange). Several factors stimulate myelopoiesis in the bone marrow to enable transition of MDSCs from hematopoietic stem cells, including granulocyte–macrophage colony-stimulating factor (GM-CSF), macrophage colony-stimulating factor (M-CSF), granulocyte colony-stimulating factor (G-CSF), stem cell factor (SCF), Interleukin-6 (IL-6), Interleukin-10 (IL-10), Interleukin-1β (IL-1β), vascular endothelial growth factor (VEGF), Hypoxia Inducible Factor 1a (HIF1a), and S100 calcium binding protein 8/9 (S100A8/9). Tumor-derived factors trigger the activation and expansion of MDSCs by activating several transcriptional factors and regulators, including JAK-STAT, NF-κB, PGE2 and IFN-γ. The recruitment of MDSCs to the TME is primarily governed by chemokines like CXCL1/2/5/6/8/12, CX3CL1, CCL1/2/3/4/5. (Right panel) MDSC Ontogeny pathway showing differentiation from hematopoietic stem cells through sustained myelopoiesis to common myeloid progenitors and granulocyte-macrophage progenitors, followed by recruitment to peripheral tissues and activation in the tumor microenvironment to form distinct MDSC subsets: PMN-MDSCs, e-MDSCs, and M-MDSCs. TNF-α, (Tumor Necrosis Factor-alpha) JAK1/2, Janus Kinase 1/2; STAT, Signal Transducer and Activator of Transcription; LAP, Leukocyte Alkaline Phosphatase; C/EBPβ, CCAAT/enhancer-binding protein-β; NF-κB, Nuclear Factor-kappa B; PGE2, Prostaglandin E2; IFN-γ, Interferon-gamma, Interferon-g; IRF, Interferon Regulatory Factor; CXCL, C-X-C Motif Chemokine Ligand; CCL, C-C motif chemokine ligand. Created in BioRender. https://BioRender.com/16fqdm5.

MDSCs emerge upon persistent pathological immune activation of the myeloid cell compartment by prolonged exposure to myeloid growth factors and inflammatory signals arising from tumors, chronic infections, inflammation, or autoimmune diseases (9). In the tumor setting, MDSC ontogeny is shaped by complex interactions between tumor-derived factors and the bone marrow niche (**Figure 1**, left panel). These immature myeloid cells originate primarily from hematopoietic stem cells in the bone marrow but can also develop from myeloid progenitors in secondary lymphoid organs such as the spleen (10) (**Figure 1**, right panel). Tumor-secreted cytokines such as G-CSF, GM-CSF, and IL-6 promote the expansion of immature myeloid progenitors in the bone marrow while simultaneously blocking their differentiation into mature dendritic cells, macrophages, or granulocytes. These expanded MDSC populations are then recruited to the tumor site through chemokine gradients, where they undergo further activation and acquire enhanced immunosuppressive functions (10). According to the latest classification system, the human MDSC population comprises three distinct groups: monocytic MDSCs (M-MDSCs), polymorphonuclear MDSCs (PMN-MDSCs), and early stage MDSCs (e-MDSCs) (11). The morphological and phenotypic characteristics of M-MDSCs and PMN-MDSCs closely resemble those of monocytes and neutrophils, respectively (12). e-MDSCs are the least mature population of MDSCs that are phenotypically devoid of granulocytic and monocytic surface markers (11).

Several phenotypic markers have been established to distinguish between MDSC subtypes in humans and murine models (**Table 1**). Moreover, recent studies have advanced the identification of MDSCs by introducing novel surface and intracellular markers to distinguish MDSC subtypes from other myeloid cells across various cancers, thereby enhancing diagnostic and prognostic precision (**Table 1**). For instance, a combination of markers, including CD14, CD68, CD163, CD206, and S100A9, has been employed in immunofluorescent multiparametric assays to accurately delineate MDSCs, offering improved specificity over traditional markers (13). A CRISPR-Cas9 screen recently identified CD300ld, expressed specifically in neutrophils, as a crucial marker of tumoral PMN-MDSCs (14). Moreover, excluding CD123^+^ cells from e-MDSC populations helps to distinguish them from basophils, addressing concerns about marker specificity and improving diagnostic accuracy (15).

**Table 1:** Distinct features, phenotypic, and emerging markers of MDSC subtypes.

Feature	M-MDSC	PMN-MDSC	e-MDSC	References
Lineage	Monocyte	Neutrophil	Immature myeloid cells	(11, 12)
Phenotypic markers*	HLA-DR^–/low^ CD11b^+^, CD14^+^, CD15^–^, CD33^+^	HLA-DR^–/low^ CD11b^+^, CD14^–^, CD15^+^	HLA-DR^–/low^ CD11b^+^, CD14^–^, CD15^–^, CD3^–^, CD19^–^, CD56^–^, CD33^+^	(11, 12)
Emerging markers*	S100A9, CD84^+^, JAM1, TREM2	LOX1, CD84^+^, CD66b^+^, SPARC, CD300ld	CD25^+^, CD123^-^	(13, 14)
Phenotypic markers**	CD11b^+^, Ly6G^–^, Ly6C^high^	CD11b^+^, Ly6G^+^, Ly6C^low^	None	(11)
Emerging markers**	CD244^+^, CD36^+^	FATP2, CD36^+^	None	(13)

HLA-DR, Human Leukocyte Antigen-DR isotype; CD, Cluster of Differentiation; S100A9, S100 calcium-binding protein A9; JAM1, Junctional Adhesion Molecule 1; TREM2, Triggering Receptor Expressed on Myeloid Cells 2; LOX1, Lectin-like oxidized low-density lipoprotein receptor-1; SPARC, Secreted Protein, Acidic and Rich in Cysteine; Ly6C, Lymphocyte antigen 6 complex, locus C1; FATP2, Fatty Acid Transport Protein 2; *human; **mouse.

## MDSC-induced immunosuppression in TME

MDSCs are pivotal orchestrators of immunosuppression within the TME and deploy a repertoire of mediators and molecules to suppress key immune components, including T cells, natural killer (NK) cells, macrophages, and dendritic cells (DCs) (16). Recent studies have elucidated the molecular mechanisms underlying these interactions, highlighting their roles in tumor immune evasion and progression across cancers. By leveraging metabolic reprogramming, cytokine/chemokine signaling, and immune checkpoint molecules, MDSCs create a suppressive network that hampers antitumor immunity, making them critical targets for therapeutic interventions. MDSCs reprogram their metabolism to deplete essential nutrients and produce toxic by-products in a subset specific manner (6, 10, 17–19). PMN-MDSCs primarily suppress T cells by generating large amounts of reactive oxygen species (ROS) through NADPH oxidase 2 (NOX2) and peroxynitrite (ONOO-) (6, 10). These reactive intermediates induce nitration of T cell receptors and inhibit antigen-specific interactions, disrupting T cell responses (6, 10). In contrast, M-MDSCs rely more heavily on Arginase-1 (ARG1), indoleamine 2,3-dioxygenase (IDO), and inducible nitric oxide synthase (iNOS) to deplete L-arginine and nitric oxide (NO) production to impair receptor signaling, inhibit T cell proliferation, and induce regulatory T-cell expansion to repress T-cells (6, 10, 17–19). MDSCs also secrete immunosuppressive cytokines, such as IL-10, IL-1RA, and TGF-β, which inhibit T-cell activation, promote regulatory T-cell expansion, and polarize macrophages toward M2 phenotypes (6, 20). Chemokines such as CCL2 and CXCL8 recruit additional MDSCs and Tregs, thereby sustaining the suppressive milieu (21). MDSCs directly suppress T cells via programmed death-ligand 1 (PD-L1) expression and amino acid depletion, thereby reducing cytotoxicity and proliferation (22). MDSCs expressing PD-L1 engage PD-1 on T cells to induce anergy, whereas VISTA and TIM-3 drive anti-PD-1 resistance, and TIGIT/CD155 interactions inhibit NK cell IFN-γ across solid and hematological malignancies (6, 23, 24). MDSCs induce downregulation of the activating receptor NKG2D on both CD8+ T cells and NK cells via membrane-bound TGF-b and soluble NKG2D ligands (MICA/MICB, ULBP); and impair maturation and cross-presentation of dendritic cells via IL-10, NO, and oxidized lipid transfer (6, 25, 26). CD39 and CD73 ectonucleotidases in MDSCs generate adenosine, activating A2A/A2B receptors on T cells, NK cells, and macrophages to suppress their effector functions (27). This multifaceted crosstalk between MDSCs and other immune components promotes immune tolerance in the TME and facilitates tumor progression.

## Therapeutic strategies targeting MDSCs

### Metabolic targeting in MDSCs

**Figure 2:**
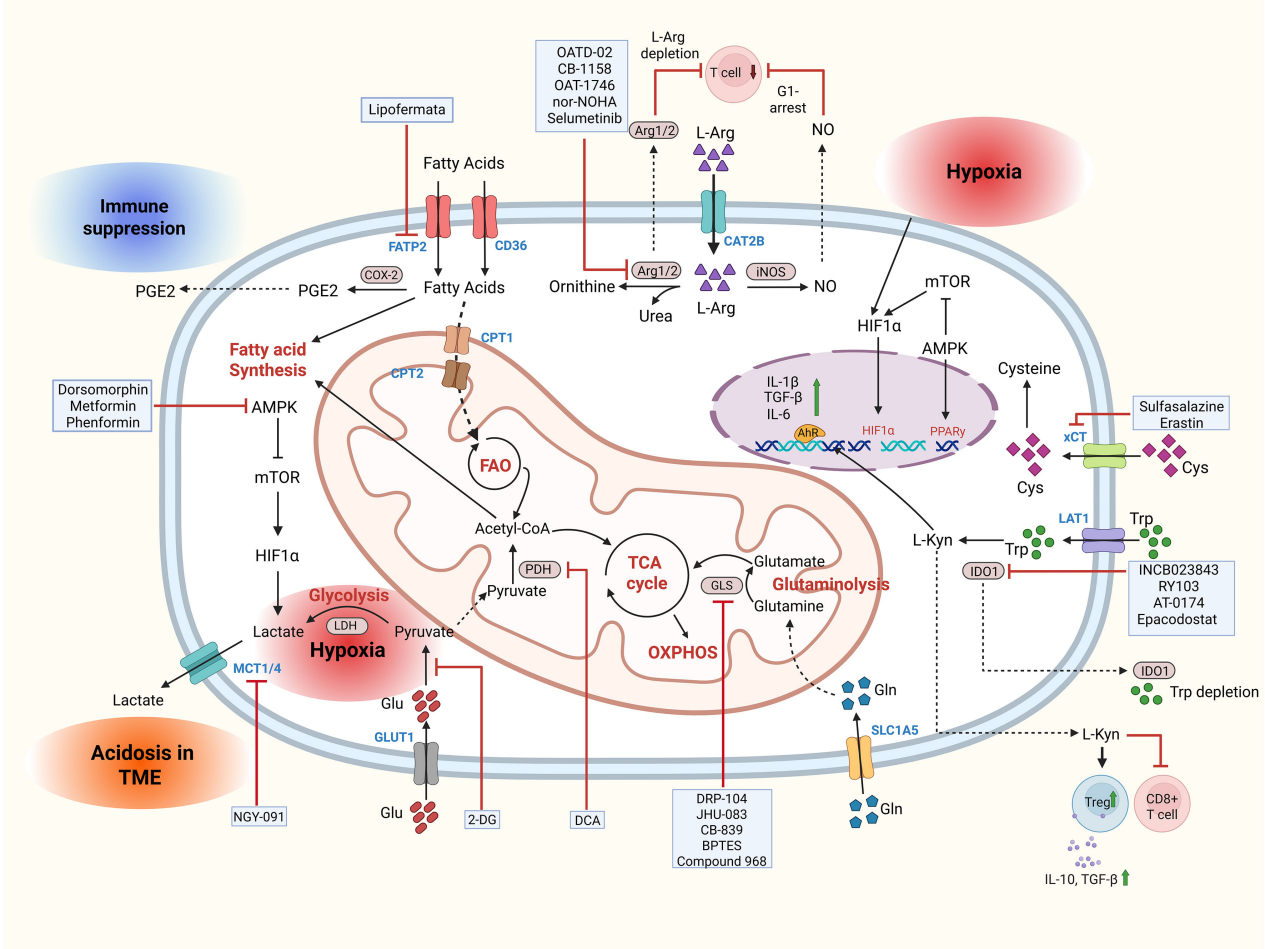
Metabolic regulation in MDSCs and list of therapeutics targeting key metabolic checkpoints in MDSCs. FAO, Fatty Acid Oxidation; Cys, Cystine; Trp, Tryptophan; L-Kyn, L-kynurenine; GLS, Glutaminase; GLUT1, Glucose Transporter 1; OXPHOS, Oxidative Phosphorylation; PDH, Pyruvate Dehydrogenase; L-Arg, L-Arginine; Gln, Glutamine; TCA cycle, Tricarboxylic Acid cycle; IDO1, Indoleamine 2,3 -dioxygenase; LAT1, L-type Amino Acid transporter 1; CAT2B, Cationic Amino Acid Transporter 2B. Created in BioRender. https://BioRender.com/1r9ubqz.

MDSCs display high metabolic reliance and undergo extensive metabolic reprogramming in the TME to adapt to harsh conditions, such as hypoxia, oxidative stress, and nutrient scarcity (6). MDSCs remarkable functional plasticity within the TME enhances their immunosuppressive and pro-tumorigenic functions in response to diverse signals from the tumors and surrounding stromal cells (28). These include molecules and enzymatic regulators involved in the metabolism of glucose, arginine, tryptophan, glutamine, cysteine, lipids, ROS, and AMP-activated protein kinase (AMPK) (28) (**Figure 2**). Targeting metabolic pathways can selectively impair their suppressive activity and expansion without broadly depleting immune cells, potentially minimizing their side effects (29). Metabolic pathways are less likely to develop rapid mutational resistance than signaling cascades because they involve enzymatic processes rather than easily mutable kinases and receptors (6). This makes metabolic reprogramming a promising therapeutic avenue to modulate MDSCs to improve anti-tumor immunity and overcome immune resistance (29).

#### Glucose metabolism

Similar to cancer cells, MDSCs undergo metabolic reprogramming characterized by a pronounced shift from OXPHOS toward aerobic glycolysis in the hypoxic, acidic, and nutrient-depleted TME (30). This glycolytic shift from OXPHOS not only fuels MDSC’s higher energy demands of MDSCs but also produces glycolytic byproducts such as lactate and ROS to create an immunosuppressive microenvironment (30). Although hyperactivation of glycolytic signaling pathways is common in MDSCs, the reliance of MDSC subsets on OXPHOS and aerobic glycolysis is heterogeneous (30). Tumor-infiltrating M-MDSCs in hypoxic TMEs generally rely more heavily on aerobic glycolysis than OXPHOS, whereas PMN-MDSCs meet their energy demands via both OXPHOS and aerobic glycolysis (22). The reliance of MDSCs on glucose and glycolytic metabolism has been extensively targeted, either by depleting glucose (e.g., 2-DG) or inhibiting glucose transporters and glycolytic enzymes such as GLUT3 or pyruvate dehydrogenases (e.g., DCA) (31, 32). Interestingly, the glycolysis inhibitor resveratrol (trans-3,4′,5-trihydroxystilbene) co-delivered with PD-L1 siRNA skews MDSCs from glycolysis to OXPHOS, thereby weakening their immunosuppressive activity and boosting anti-tumor immunity (33). Mitochondria-targeted atovaquone (Mito-ATO) leverages the FDA-approved anti-malarial drug atovaquone’s electron-transport inhibitory activity to selectively impair the bioenergetics of PMN-MDSCs (34). Mito-ATO, with its conjugated lipophilic triphenyl phosphonium (TPP) moiety, accumulates within mitochondria and blocks mitochondrial complex I and glycolytic pathways in PMN-MDSCs, the predominant MDSC subset in the murine model, leading to reduced MDSC viability, diminished ARG-1 and ROS production, and restoration of T cell-mediated anti-tumor immunity (34).

Increased glycolytic flux and glucose uptake lead to excess lactate production that is transported across membranes via monocarboxylate transporters 1 and 4 (MCT1 and MCT4), thereby allowing the cells to prevent intracellular acidification and maintain high glycolytic activity (35, 36). Excess lactate induces the activation of MDSCs and facilitates their immunosuppressive and tumor-promoting activities, which is circumvented by blocking lactate transport by NGY-091, a dual MCT1/4 inhibitor (37). While these strategies are mostly preclinical, clinical translation of these strategies is limited due to off-target effects on other immune cells residing in the TME that rely on glycolysis as an energy source.

#### AMPK

AMP-activated protein kinase alpha (AMPKα) is a crucial cellular energy sensor and metabolic regulator that maintains energy homeostasis, with controversial roles in modulating MDSC biology (38). MDSCs isolated from LLC mouse tumors and advanced/high-grade serous ovarian cancer patients display increased AMPKα activation driven by STAT5 and cancer-derived GM-CSF (38). Genetic deletion or pharmacological blockade of AMPKα by Dorsomorphin (Compound C) diminishes MDSC immunosuppression by lowering ARG1 and increasing Nos2 levels (38). Conversely, the type 2 diabetes medication metformin activates AMPKα and hinders the immunosuppressive potential of CD39^+^CD73^+^MDSCs by targeting their adenosinergic activity, thereby improving anti-tumor immunity and overall survival in ovarian cancer patients with diabetes (39). In another study, metformin enhanced AMPK phosphorylation to induce DACH1, which inhibited NF-κB signaling and restricted PMN-MDSC migration into tumors (40). These findings underscore the context-dependent and ambiguous effects of AMPK signaling on MDSC function.

#### Amino acid metabolism

Myeloid-derived suppressor cells (MDSCs) exploit multiple amino acid metabolic pathways to establish and sustain immunosuppression in the tumor microenvironment (7). Three pathways, arginine, tryptophan, and glutamine/cysteine metabolism, are particularly important because they directly restrict T-cell function, promote MDSC survival, drive MDSC differentiation, and are amenable to pharmacological targeting (41). MDSC-mediated depletion of extracellular l-arginine is a canonical mechanism of T cell suppression. Central to this process is the elevated expression of arginase 1 (ARG1) by MDSCs, which catalyzes the hydrolysis of l-arginine to ornithine and urea, thereby depleting extracellular arginine and effectively impairing T cell responses (17). Pharmacological inhibition of ARG1 and, in some cases, ARG2 using small molecules such as CB-1158, OAT-1746, and OATD-02 has consistently demonstrated restoration of T cell proliferation and enhanced antitumor immunity in preclinical models, often synergizing with checkpoint inhibitors or adoptive cell therapies (42–45). These inhibitors elevate intratumoral arginine levels, reduce MDSC frequencies, and facilitate greater CD8^+^ T-cell infiltration, thereby creating a more immune-permissive microenvironment (42–44). Synthetic arginase inhibitors (e.g., Nor-NOHA) also show the potential to modulate ARG1 expression and MDSC function to improve immunotherapy outcomes (46). Although the suppressive function of ARG1 is well established, studies have shown heterogeneity in its expression and impact across tumor types and microenvironments. Some data indicate that ARG1 activity correlates with enhanced MDSC-mediated immunosuppression and poorer outcomes in cancers, such as non-small cell lung cancer (NSCLC) and colorectal cancer, where ARG1^+^ MDSCs promote immune escape and tumor progression (47, 48). Conversely, other reports suggest that ARG1 expression may be inducible rather than constitutive, with variable effects in models such as melanoma and lymphoma (49). This underscores the metabolic and functional plasticity of MDSCs depending on the tumor context. Importantly, MDSCs can suppress T cells through arginase-independent pathways, including cytokine-mediated induction of ARG1 and other immunosuppressive factors, highlighting the varied metabolic dependencies of MDSCs across different tumor microenvironments (49). This complexity emphasizes the need to consider MDSC heterogeneity and metabolic flexibility when designing targeted therapies.

Tryptophan (Trp) catabolism through the kynurenine (L-Kyn) pathway is another major immunoregulatory axis employed by MDSCs to exert immunosuppression in the TME (18). The primary rate-limiting enzyme, indoleamine 2,3-dioxygenase 1 (IDO1), which converts Trp to L-Kyn, is frequently expressed in tumor and circulating MDSCs (18). L-Kyn activates the aryl hydrocarbon receptor (AhR) and general control nonderepressible 2 (GCN2) stress kinase, which drives the expansion and survival of MDSCs in tumors, while suppressing anti-tumor immunity via amino acid starvation in effector T cells and tolerogenic transcriptional programs (18). IDO1 is frequently expressed in tumor-associated MDSCs and can promote regulatory B/T cell differentiation and neovascularization via downstream GCN2 signaling (50, 51). Pharmacological IDO1 inhibitors (e.g., INCB023843 and RY103) lower MDSC burden and restore CD8 + T-cell infiltration in preclinical settings (52, 53). Notably, RY103 showed efficacy in blocking IDO1-GCN2–mediated immunosuppression in glioma models, but failed to impact MDSCs, possibly due to limited infiltration of MDSCs into the glioma TME (54). Epacadostat, a highly potent IDO1 inhibitor, despite its initial promise in preclinical settings, demonstrated no clinical benefit in combination with the PD-1 inhibitor pembrolizumab in a large Phase III trial (NCT02752074), possibly due to compensatory mechanisms such as upregulation of IDO2 and tryptophan-2,3-dioxygenase (TDO), which sustains immunosuppressive kynurenine production (55). Emerging strategies following the failure of epacadostat in clinical trials include a novel epacadostat nanovesicle therapeutic platform (Epacasome) that, in combination with α-PD-1 therapy, enhanced the reduction of PMN-MDSCs compared to free epacadostat, potentially offering a more robust delivery of epacadostat to counter tumor immunosuppression (56). Another strategy aims to simultaneously inhibit IDO1 and TDO2 with AT-0174 in platinum-refractory lung tumors, which utilize both enzymes for survival and immune evasion (57).

Glutamine (Gln) metabolism is critical for myelopoiesis, differentiation from immature precursors, and immunosuppressive functions of MDSCs within the TME (58). Glutamine is converted by glutaminase (GLS) into glutamate and subsequently into α-ketoglutarate (α-KG), which fuels the tricarboxylic acid (TCA) cycle (59). MDSCs are marked by elevated glutaminolytic flux compared to other myeloid cells, as they facilitate the upregulation of immunosuppressive mediators (ARG1, iNOS, and PD-L1) via HIF-1α-mTORC1 activation (60). Moreover, glutathione, a derivative of glutamine, protects MDSCs from oxidative stress within the hypoxic TME by improving their ROS neutralizing ability (61). Hence, targeting glutamine metabolism has emerged as a promising strategy for disrupting MDSC-mediated immunosuppression. DON (6-Diazo-5-oxo-L-norleucine), a glutamine antagonist with anticancer activity, has limited clinical efficacy owing to its significant toxicity and broad and non-selective inhibition of glutamine-dependent enzymes (62). Consequently, prodrugs of DON (e.g., JHU083, DRP-104, JHU395) with more specific targeted inhibition of glutamine pathways have emerged (60). JHU083 selectively inhibits the production of MDSCs, blocks their recruitment, reprograms them toward a proinflammatory phenotype, and shows synergy with checkpoint blockade therapy to enhance anti-tumor immunity (60, 63). Moreover, the selective bio-activation property within tumors and inactivation in the gastrointestinal tissue of DRP-104 circumvents the toxicities linked to DON, while also lowering MDSCs in the TME (64, 65). Pharmacological inhibition of glutaminase with CB-839 (telaglenastat), BPTES (bis-2-(5-phenylacetamido-1,2,4-thiadiazol-2-yl) ethyl sulfide), and compound 968 has been studied across various tumor types and in combinatorial approaches; however, their direct impact on MDSCs depends on the tumor type, drug dosing, and compensatory pathways, which remain to be examined (66–70).

MDSCs import extracellular cystine by exploiting the xCT (SLC7A11) antiporter and generate intracellular cysteine to support the redox balance and immunosuppressive functions (71). Pharmacological inhibitors of xCT (sulfasalazine, erastin) reduce tumor MDSC infiltration and metastatic spread in preclinical models, although their effects on primary tumor growth can be variable (71). xCT blockade therefore represents a way to perturb MDSC redox metabolism and trafficking but may need to be combined with complementary approaches to achieve durable tumor control.

#### Lipid metabolism

Although MDSCs predominantly rely on glycolysis and OXPHOS as a bioenergetic source, lipid uptake and catabolism via fatty acid oxidation (FAO) are now recognized as central metabolic programs that enable MDSCs to survive, expand, and exert potent immunosuppressive functions in the TME (72). Tumor-derived factors and enriched lipid content in the TME upregulate the expression of lipid transporters (members of the fatty acid transport protein FATP1/2/3/6, Lrp1) and enzymes Cyclooxygenase-2 (COX-2) that regulate fatty acid intake and fatty acid oxidation (FAO) (72, 73). Enhanced lipid-centric adaptation results in a metabolic switch, shifting MDSCs away from glycolytic metabolism toward FAO to ensure an ample supply of energy intermediates and bioactive lipid mediators to potentiate their immunosuppressive activity and longevity (72).

FATP-2, selectively expressed on PMN-MDSCs, serves as both a gatekeeper for fatty acid transport and regulator of long-chain fatty acid metabolism (73). FATP2 is an important therapeutic target in PMN-MDSCs that mediates immunosuppression by facilitating arachidonic acid (AA) uptake and prostaglandin E_2_ (PGE2) synthesis via cyclooxygenase-2 (COX-2), which promotes immunosuppression and expansion (74). Genetic deletion or pharmacological inhibition of FATP2 by lipofermata reduces PMN-MDSCs across tumors by reducing PGE2 synthesis, lowering ROS release and lipid accumulation, increasing CD8^+^ T cell infiltration into tumors, and synergizing with immune checkpoint blockade (74, 75).

Future studies should examine the long-term impact of FATP2 inhibition on immune homeostasis and potential compensatory mechanisms within the tumor microenvironment. PGE2 produced by COX-2 in tumors mediates immunosuppression of MDSCs via E-prostanoid receptor type 2 (EP2) and EP4 (76). Targeting PGE2 signaling via EP4 selective inhibitor MF-766 lowered PMN-MDSC infiltration into the TME, showing synergy with anti-PD-1 therapy (76). In addition to lipid uptake, tumorigenesis induced chronic stress activates the β-adrenergic receptor (β2-AR) in MDSCs to enhance FAO and upregulate the mitochondrial fatty acid transporter CPT1A, thereby reinforcing their PGE2 mediated immunosuppressive capacity (77, 78). β2-AR activated signaling also promotes mitochondrial fitness and survival of MDSCs by regulating ATP synthesis and itaconate metabolism, making it a candidate for metabolic targeting in MDSCs (61).

Several other lipid metabolism pathways are currently being investigated in MDSCs, including Squalene epoxidase (SQLE), N-acylsphingosine amidohydrolase (ASAH2), and TNF-α-induced protein 8-like 2 (TIPE2) (79–81). SQLE is a rate-limiting enzyme in the cholesterol biosynthesis pathway that converts squalene to 2,3-oxidosqualene, ultimately driving cellular cholesterol accumulation (79). Cholesterol enrichment in MDSCs enhances immunosuppression by upregulating the production of ARG1, iNOS, and PD-L1 clusters (82). Genetic knockout or pharmacological inhibition of SQLE in tumor cells reduces cholesterol levels in MDSCs, diminishes their suppressive capacity, and restores the antitumor activity of anti-PD-1 antibodies *in vivo* (83, 84). N-acyl sphingosine amidohydrolase (ASAH2) is a neutral ceramidase with marked upregulation in tumor-infiltrating MDSCs in colon carcinoma and promotes the survival of MDSCs by regulating sphingolipid metabolism, conferring resistance to ferroptotic cell death (80). Targeting ASAH2 with a selective inhibitor NC06 (7-chloro-2-(3-chloroanilino)pyrano[3,4-e][1,3]oxazine-4,5-dione) induces ferroptosis in MDSCs by suppressing lipid ROS production, consequently inducing cell death and reducing MDSC accumulation (80). TNF-α-induced protein 8-like 2 (TIPE2), a phospholipid transfer protein, is upregulated in MDSCs upon tumoral ROS exposure in the TME, and regulates ferroptosis-induced immunosuppression in MDSCs by accumulating phospholipids and generating lipid ROS (85). Importantly, TIPE2 inhibition exhibits potent synergy in combination with ferroptosis induction and anti-PD-L1 therapy *in vivo* (85). Depleting TIPE2 also shifts pro-tumoral MDSCs toward an anti-tumoral phenotype by modulating their immunosuppressive activity, consequently lowering the tumor burden preclinically (81).

### Direct depletion of MDSCs

**Figure 3:**
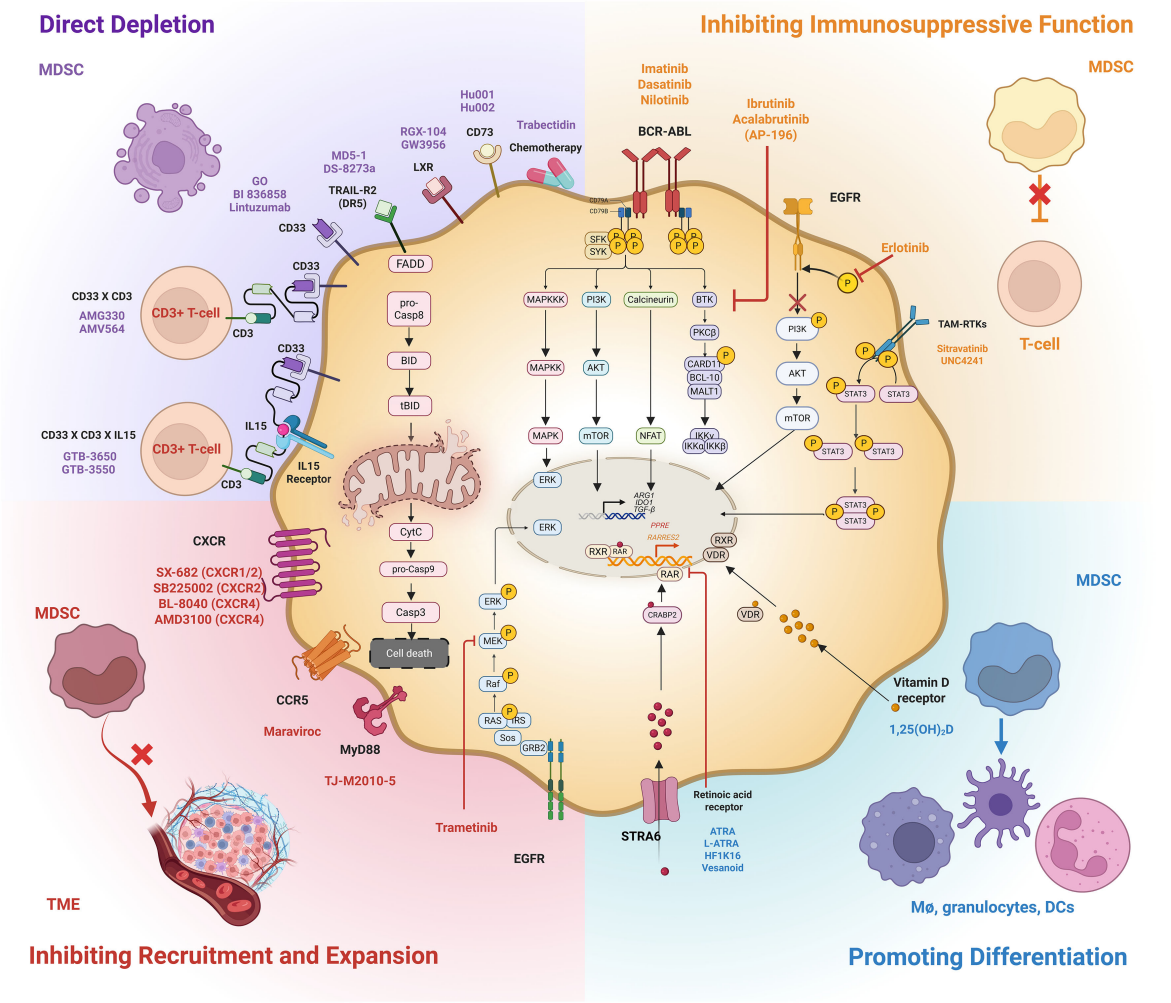
Non-metabolic therapeutic targets in MDSCs and list of therapeutics targeting key immune checkpoints in MDSCs. LXR, Liver X Receptor; TRAIL-R2, TNF-related apoptosis-inducing ligand (TRAIL) receptor 2; TAM-RTK, TYRO3, AXL and MERTK family of receptor tyrosine kinases; EGFR, Epidermal Growth Factor Receptor; BTK, Bruton’s Tyrosine Kinase; CCR5, Chemokine Receptor type 5; MEK1/2, Mitogen-Activated Protein Kinase 1/2; MyD88, Myeloid Differentiation Factor 88. Created in BioRender. https://BioRender.com/k7og38q.

MDSCs can be selectively depleted using various antibodies targeting surface markers and death receptors, thereby restoring effective anti-tumor immunity. Several monoclonal antibodies targeting distinct MDSC surface markers have been developed to deplete MDSCs and restore T cell function (**Figure 3**). Among these, monoclonal antibodies that target CD33, a hallmark of M-MDSCs, have been extensively studied. Anti-CD33 monoclonal antibody drug conjugates, such as gemtuzumab ozogamicin, primarily mediate direct MDSC depletion through antibody-dependent cellular cytotoxicity, leading to enhanced T cell and CAR-T cell function (86).

Additionally, non-conjugated monoclonal antibodies (e.g., lintuzumab or BI 836858) inhibit MDSC differentiation and immunosuppression by disrupting SHP-1/2 phosphatase activity (87, 88). Although BI 836858 reduced MDSCs in preclinical studies, it failed to lower overall MDSC numbers or activate NK cells in myelodysplastic syndrome (MDS)/acute myeloid leukemia (AML) patients, leading to the termination of clinical development (88).

Bi-specific antibodies, such as AMG 330 and AMV564, link MDSCs to T cells by simultaneously binding CD33 and CD3, effectively re-engaging cytotoxic T cells to eliminate CD33^+^ MDSCs and tumor cells (89, 90). This dual mechanism might elicit more potent and precise immune responses than off-target effects on normal myeloid cells upon administration of monoclonal antibodies.

Furthermore, tri-specific antibodies (e.g., GTB-3650 and GTB-3550) combining CD33 targeting with IL-15 activation are under clinical investigation (NCT06594445) (**Table 2**), representing an innovative strategy to synergize direct depletion with immune activation (91, 92).

**Table 2:** Clinical trials targeting MDSCs in solid and hematologic malignancies.

Compound (target)	Clinical phase (status)	Tumor	Combination partner(s)	Clinical trial No.	References
HF1K16 (ATRA)	Phase I (ongoing)	Solid tumors	NA	NCT05388487	(150)
VESANOID (ATRA)	Phase II (completed)	Melanoma	Pembrolizumab	NCT03200847	(146)
SX-682 (CXCR1/2)	Phase I (ongoing)	Melanoma	Pembrolizumab	NCT03161431	(121)
SX-682 (CXCR1/2)	Phase I (ongoing)	PDAC	Nivolumab	NCT04477343	(122)
GTB-3650 (CD16/IL-15/CD33)	Phase I (ongoing)	MDS, AML	NA	NCT06594445	(92)
Motixafortide/BL-8040 (CXCR4)	Phase II (completed)	PDAC	Pembrolizumab/Onivyde/LV/5-FU	NCT02826486	(126)
RGX-104 (LXR agonism)	Phase I (completed)	NSCLC, SCLC, Endometrial Ca.	Nivolumab/Ipilimumab Docetaxel Pembrolizumab Carboplatin/Pemetrexed	NCT02922764	(166)
Ibrutinib (BTK)	Phase III (completed)	CLL, SLL	NA	NCT01724346	(109)
ACP-196 (BTK)	Phase II (completed)	Pancreatic Ca.	Pembrolizumab	NCT02362048	(107)
Ibrutinib (BTK)	Phase I (completed)	Metastatic Solid Tumors	Nivolumab	NCT03525925	(110)
Bezafibrate (PPARγ)	Phase I (ongoing)	NSCLC	Nivolumab	UMIN000029854	(163)
OATD-02 (Arginase 1/2)	Phase I (ongoing)	RCC, Ovarian Ca., CRC, Pancreatic Ca.,	NA	NCT05759923	(167)
INCB001158 (Arginase)	Phase I (completed)	NSCLC, Melanoma, Urothelial Ca., MSI/MSS- CRC, Gastric Ca., HNSCC, Mesothelioma	Pembrolizumab	NCT02903914	(168)
Tadalafil (PDE5)	Phase I (completed)	HNSCC	Anti-MUC1 Vaccine	NCT02544880	(169)

PDAC, Pancreatic Adenocarcinoma; AML, Acute Myeloid Leukemia; MDS, Myelodysplastic syndrome; NSCLC, Non-Small Cell Lung Cancer; SCLC, Small Cell Lung Cancer; CLL, Chronic Lymphoblastic Leukemia; SLL, Small Lymphoblastic Leukemia; RCC, Renal Cell Carcinoma; CRC, Colorectal Cancer, HNSCC, Head and Neck Squamous Cell Carcinoma; Ca, Carcinoma; MSI, Microsatellite Instability; MSS, Microsatellite Stable; LV, Leucovorin; 5-FU, 5-Fluorouracil.

Beyond CD33 targeting, agonistic antibodies against Death Receptor 5 (DR5, also known as TRAIL-R2), such as MD5-1, selectively induce apoptosis in MDSCs by activating the extrinsic apoptotic pathway. This targeted depletion of MDSCs enhances antitumor immunity by increasing CD8^+^ T cell infiltration and activation in tumors (93). Similarly, antibodies blocking immunosuppressive pathways mediated by MDSC surface molecules, such as CD73 (e.g., Hu001 and Hu002) and CD200, reduce MDSC accumulation and function, further relieving immune suppression (94, 95).

The liver-X nuclear receptor (LXR), a key regulator of lipid metabolism and cholesterol transport, has also emerged as a therapeutic target in MDSCs (96). LXR agonists, such as RGX-104 and GW3965, reduce MDSC levels by activating the LXR/ApoE axis and reversing cholesterol transport, thereby improving the anti-tumor response of T cells in preclinical and clinical settings (NCT02922764) in various treatment-refractory tumors, resensitizing patients to anti-PD1 therapy (97). A recent study using a myeloid cell-depleting chemotherapeutic agent Trabectedin in combination with IL-12 therapy was shown to significantly deplete splenic MDSCs and restore NK cell cytotoxic function in a triple negative breast cancer (TNBC) model (98).

Combining MDSC-targeted antibodies with CAR-T cell therapies or immune checkpoint blockades holds great promise by simultaneously reducing immunosuppressive barriers and potentiating T cell-mediated cytotoxicity. This integrated approach could overcome resistance mechanisms and improve clinical outcomes in both hematologic and solid tumors. Thus, elucidating the distinct mechanisms of antibody action and maximizing combinatorial regimens is critical for advancing MDSC-targeting immunotherapies.

### Inhibiting immunosuppressive function of MDSCs

A growing body of evidence highlights the pivotal role of receptor tyrosine kinases in driving MDSC accumulation and function within the TME (99, 100). For example, AXL signaling concurrently upregulates IL-6 and GM-CSF production, thereby promoting MDSC accumulation and expansion (99, 100). Genetic or pharmacological AXL depletion causes a significant reduction in G-CSF levels and impairs the differentiation and recruitment of PMN-MDSCs (99, 100). Beyond AXL, TYRO3, AXL, and MERTK transmembrane receptor tyrosine kinases (TAM RTKs) play crucial roles in innate immunity and are frequently overexpressed in several solid malignancies (101). Among TAM receptor tyrosine kinases, MERTK^+^ MDSCs are significantly enriched across all three MDSC subtypes in patients with metastatic melanoma (102).

Hence, MERTK^+^ MDSCs may serve as a potential biomarker for malignancy and as a predictive indicator of therapeutic response to TAM RTK inhibitors. Moreover, genetic depletion or pharmacological inhibition of TAM RTKs by UNC4241 mitigated MDSC-mediated immunosuppression, reduced tumor growth, increased CD8^+^ T cell infiltration at the tumor site, and enhanced the effectiveness of anti-PD-1 therapy (102). Sitravatinib, a broad-spectrum TAM RTK inhibitor, reduced M-MDSC frequencies, decreased PD-L1 + M-MDSCs, and lowered Arg-1 and IL-4 levels in murine lung carcinoma models, thereby enhancing immune checkpoint blockade (103).

Bruton’s tyrosine kinase (BTKs) is an essential regulator of B-cell proliferation and survival (104). In addition to their role in B cells, BTKs are expressed in tumoral MDSCs and contribute to their maturation and functional activity (105). Ibrutinib, a BTK inhibitor, reduces MDSC generation and diminishes immunosuppressive effects *in vitro*, including decreased NO production, impaired migration, and lowered IDO expression (105). Ibrutinib treatment reduces PMN-MDSC levels in chronic lymphocytic leukemia (CLL) within three months, while leaving M-MDSC unchanged (106). Similarly, acalabrutinib (AP-196), a third-generation BTK inhibitor in combination with anti-PD-1 therapy in a Phase II trial (NCT02362048), displayed sustained and selective reduction in PMN-MDSCs while sparing M-MDSCs (107). The discrepancy in PMN-MDSC and M-MDSC levels upon BTK inhibition indicates that the subtypes have distinct sensitivities to BTK inhibition, warranting further evaluation of BTK signaling in MDSC subsets (106, 107). Ibrutinib enhances the efficacy of anti-PD-L1 therapy in murine breast cancer and melanoma models (105, 108) Follow up evaluation of CLL patients from a Phase III clinical trial (NCT01724346) indicated the long-term impact of ibrutinib treatment in reducing MDSC levels to healthy donor range (109). Ibrutinib treatment reduces the expression of several chemokines involved in MDSC recruitment, downregulates genes conferring MDSC-suppressive functions, and reduces MDSC interaction with T cells, thereby improving T cell proliferation in metastatic solid tumors when combined with the anti-PD-1 antibody nivolumab (110). Notably, gene analysis revealed increased expression of MHC class II antigen presentation genes on MDSCs in patients with clinical benefit, indicating possible differentiation and maturation of MDSCs upon BTK inhibition (110).

Tyrosine kinase inhibitors (TKIs) directed against BCR-ABL, including first-line TKI imatinib and second-generation TKIs dasatinib and nilotinib directed against the Philadelphia chromosome fusion gene BCR-ABL, effectively reduced PMN-MDSC levels in CML and Ph+ ALL patients; however, dasatinib-treated patients had reduced M-MDSC levels, especially when combined with anti-PD1 therapy (111–114).

Beyond lineage-specific RTKs, broad-spectrum kinase inhibitors such as cabozantinib, erlotinib, sorafenib, and anlotinib exert multifaceted approaches to target MDSC (115–118) For example, cabozantinib combined with anti-HER2 immunotherapy reprograms MDSC by suppressing ARG1 and reducing their frequencies (115). Similarly, targeting EGFR with erlotinib diminishes MDSC abundance in pancreatic cancer models, enhancing CD8^+^ T cell infiltration and immunotherapy responses (117). In contrast, sorafenib paradoxically enhances MDSC accumulation and immunosuppression by promoting FAO via PPARα activation and upregulating CCR2 signaling, with the effects reversed upon CCR2 blockade or inhibition of FAO/PPARα (116). Anlotinib, when combined with anti-PD1 therapy and radiotherapy, decreases MDSCs and ARG1 expression, boosting CD8+ T cell infiltration and IFNγ levels preclinically (118).

### Inhibiting recruitment and expansion of MDSCs

The recruitment and accumulation of MDSCs in the TME create an immunosuppressive microenvironment, leading to poor clinical outcomes and therapy resistance. Therapeutic strategies to interrupt MDSC trafficking to the TME offer a unique approach, with particular attention given to chemokines and chemokine receptors, cytokines, and intracellular signaling pathways.

Chemokines are essential for recruiting MDSCs to the TME, and their blockade shows significant therapeutic potential (119). Pharmacological inhibition of chemokine-mediated recruitment using CXCR1/2 inhibitors (SX-682, SB265610), CXCR4 inhibitors (AMD3100, BL-8040), or partial CXCR4 agonists (TFF2-MSA) curtails the accumulation of MDSCs and restores anti-tumor immunity by restoring NK and T cell function in preclinical models, orchestrating synergistic therapeutic outcomes when combined with immune checkpoint blockade therapies in clinical trials (120–128). CCR5 and its ligands (CCL3, CCL4, CCL5) facilitate PMN-MDSC proliferation and recruitment (129). Blocking CCR5 and its ligands with a neutralizing fusion CCR5 protein (mCCR5–Ig) or the CCR5 antagonist maraviroc impairs MDSC migration (129, 130). Other factors such as S100A4 and S100A9 drive MDSC expansion and accumulation through chemokine regulation. Targeting these molecules or their downstream receptors reduces MDSC recruitment and enhances response to immunotherapy (131–133).

Inhibiting cytokines such as macrophage migration inhibitory factor 2 (MIF2) with MIF inhibitors sulforaphane and ibudilast, VEGF via thymosin α1 or its analog thymalfasin, or genetic ablation of cytokine inducing factors such as Yes-associated protein 1 (YAP1) reduces MDSC expansion and migration to the TME preclinically (134–139).

Downstream signaling pathways of the MAPK cascade, including mitogen-activated protein kinase kinase 1 and 2 (MEK1/2) of the MAPK cascade regulate CCL2 expression and MDSC recruitment. Inhibition of MEK1/2 by trametinib reduces CCL2 levels, limits MDSC accumulation, and improves outcomes, particularly when combined with anti-MDSC agents or checkpoint inhibitors (140). The adaptor protein myeloid differentiation factor 88 (MyD88) regulates several factors involved in MDSC expansion, including G-CSF, IL-6, and TGF-β. Blocking MyD88 with TJ-M2010–5 reduces these factors, thereby limiting the differentiation of myeloid cells into suppressive phenotypes (141). More recently, transmembrane BAX inhibitor motif-containing 1 (TMBIM1), a negative regulator of apoptosis, has emerged as a therapeutic target. TMBIM1 promotes the transcription of both PD-L1 and CCL2 through its interaction with YBX1 in pancreatic cancer, resulting in increased MDSC infiltration (142).

Cell surface receptors such as triggering receptors expressed on myeloid cell 1 (TREM1) are expressed by MDSC subsets and regulate cytokine signaling within the TME (143). Inhibition of TREM1 by VJDT reduces immunosuppressive activity and MDSC recruitment (144). Immunotherapy-activated CD8 + TILs promote PMN-MDSC infiltration by upregulating lipocalin-2 (LCN2) via fatty acid synthesis and NF-κB activation (145). Agents such as glucagon-like peptide 1 (GLP1) restrict LCN2 production and PMN-MDSC infiltration and restore anti-tumor immunity in mouse cancer models (145).

### Promoting MDSC differentiation

Another therapeutic strategy to counter MDSCs is to promote their differentiation into mature, non-suppressive myeloid cells, such as dendritic cells, macrophages, or granulocytes (146). Among the most extensively studied agents is all-trans-retinoic acid (ATRA), a naturally occurring retinoid and well-established standard of care for acute promyelocytic leukemia (146). ATRA promotes MDSC maturation by activating ERK1/2-MAPK signaling and lowering the expression of immunosuppressive genes NOX1, IL-10, TGF-β, IDO, and PD-L1 (147, 148). Improved formulations of ATRA, such as liposome-encapsulated ATRA (L-ATRA), showed better bioavailability than free ATRA and significantly reduced tumor-infiltrating M-MDSCs (149). Furthermore, *in vitro* studies demonstrate that L-ATRA promotes the expression of myeloid differentiation markers (CD11b, CD11c), inducing a dose-dependent reduction in HLA-DR^-^CD33^+^ MDSCs, and increasing the proportion of HLA-DR^+^CD11c^+^ DCs (149). Another ATRA formulation (Vesanoid) in combination with the anti-PD-1 monoclonal antibody pembrolizumab and the anti-CTLA-4 immune checkpoint inhibitor (ICI) ipilimumab significantly reduced circulating MDSCs in melanoma responders, which correlated with increased ATRA-induced differentiation of MDSCs into mature HLA-DR+ myeloid cells (146, 148).

HF1K16, another liposomal formulation comprising a lipid bilayer and ATRA that is currently undergoing Phase I trials for solid tumors, effectively reduced MDSCs in dose escalation studies (150). Apart from ATRA, Vitamin D analogs such as 1α, 25-Dihydroxyvitamin D (1,25(OH)_2_D) act through the vitamin D receptor (VDR) to modulate MDSC differentiation (151). They reduce the immunosuppressive functions of MDSCs and promote their maturation into non-suppressive cells, such as DCs or macrophages. This enhances antitumor immunity by alleviating MDSC-mediated T-cell suppression (151). p53 plays a multifaceted role in TME, governing immune cell regulation, differentiation, and immunosuppression (152). Activation of p53 by the pharmacological MDM2 inhibitor nutlin-3a skews MDSCs to Ly6C^+^ CD103^+^ dendritic cells preclinically (153). Toll-like receptors (TLRs) have been shown to mediate MDSC differentiation into antigen presenting cells (154). Activation of the TLR1/2 signaling pathway by the TLR1/2 agonist bacterial lipoprotein (BLP) inhibits M-MDSC-mediated immunosuppression by promoting their differentiation into M1 macrophages in a murine model of lung cancer, marked by decreased ARG1 and CD206 levels (154). Similarly, activation of TLR2 signaling with Pam3CSK4, a TLR2 agonist, induced differentiation of MDSCs into macrophages and DCs via Runx1 in HCC tumor models (155). Moreover, the TLR7/8 agonist R848 resensitized colorectal cancer cells to oxaliplatin chemotherapy by reversing oxaliplatin-suppressed differentiation of MDSCs into M1 macrophages, highlighting R848 as an immuno-active adjuvant in chemo-refractory colorectal cancer (156).

## MDSC targeting with immune checkpoint blockade

Immune checkpoint blockade (ICB) has been a promising avenue in immunotherapy in recent years; however, favorable treatment outcomes are often limited by the development of therapy resistance and a lack of durable response (157). Clinically, high intratumoral and circulating levels of MDSCs have been correlated with poor response to ICIs across multiple cancer types, including melanoma and non-small cell lung cancer (158). Anti-PD-1 therapy reduces MDSC infiltration in ICB-sensitive tumor models but fails in refractory tumors, highlighting the need for combinatorial strategies to sensitize resistant tumors and enhance ICB efficacy (159).

PD-L1-overexpressing NSCLC cells have been shown to enhance both the migratory capacity and immunosuppressive function of MDSCs by activating JAK2/STAT3 signaling, contributing to ICB resistance (160). Moreover, targeting the G-protein-coupled receptor GPR84 expressed on MDSCs with the selective GPR84 inhibitor GLPG1205 restores sensitivity to anti-PD-1 therapy in melanoma, lung cancer, and esophageal cancer models, highlighting MDSCs as a pivotal barrier to effective immunotherapy and an attractive adjunctive target to sensitize ICB-resistant tumors to ICIs (161). Treatment with artemisinin, an anti-malarial drug with immune-regulatory and anti-tumor effects, synergizes with anti-PD-L1 therapy in melanoma tumor models, inhibiting MDSC immunosuppression and accumulation and promoting T cell anti-tumor effects (162). Bezafibrate (BEZ), a peroxisome proliferator-activated receptor (PPAR) agonist with mitochondrial complex I/III inhibitory activity, displays potent synergism with anti-PD-1 therapy (163, 164, 166–168). More recently, an analog of BEZ (mito-BEZ) with increased mitochondrial localization significantly reduced both PMN-MDSCs and M-MDSCs, while increasing tumor infiltration of CD8^+^ T cells, highlighting BEZ as a potential combinatorial therapy with ICB to target MDSC-mediated immunosuppression on T cells (165). CD100ld, an emerging PMN-MDSC marker, facilitates their recruitment to tumors and T cell suppression (14). Blocking CD100ld in combination with anti-PD-1 treatment has a pronounced synergistic effect on tumors, suggesting a PMN-MDSC-specific approach for combinatorial immunotherapy (14).

Prokineticin 2 (BV8) is a key mediator in the recruitment of MDSCs to tumors (169, 170). Anti-BV8 treatment in preclinical models of lung, breast, and renal carcinomas reduces PMN-MDSCs by inhibiting their recruitment and promoting T cell activation, thereby sensitizing anti-PD-1-resistant tumors to anti-PD-1 therapy (170). Notably, MDSC-depleting anti-DR5 antibody MD5–1 synergizes with immune checkpoint inhibitors such as anti-PD-L1, depletes M-MDSCs with high DR5 expression via TRAIL-mediated apoptosis, and reduces tumor infiltration without affecting T cells or DCs, leading to significantly improved tumor suppression and durable immunological memory in syngeneic models of gastric and colon cancer (93). S-1, an oral 5-FU formulation, targets MDSCs by downregulating key immunosuppressive recruiters, thereby inhibiting MDSC accumulation in spleen/tumors without directly affecting MDSC survival/differentiation (171). Moreover, in AB1-HA mesothelioma and LLC lung models, S-1 suppresses tumor progression and MDSC infiltration, synergizes with anti-PD-1 to enhance CD8^+^ infiltration, and overcomes immunosuppression, supporting an avenue for combinatorial immunotherapy in thoracic cancers (171). Dual inhibition of CD39 (an adenosine-producing ectonucleotidase) and VISTA (a checkpoint ligand on MDSCs) reduces MDSC infiltration by 40–50% in syngeneic models, downregulating suppressive mediators, such as TGF-β and IL-10 (172). This strategy reverses CD8^+^ T-cell exhaustion, enhances IFN-γ and granzyme B levels, and synergizes with anti-PD-1 (172). By remodeling the TME to promote T-cell activity, the CD39/VISTA blockade offers a promising approach to overcome resistance to ICB and radiotherapy (172). Tyrosine kinase with Ig and EGF (epidermal growth factor) homology domain 2 (TIE-2), a receptor for the proangiogenic factor angiopoietin-2, is expressed on circulating m-MDSCs in melanoma patients, where TIE-2^+^ M-MDSCs overexpress immunosuppressive mediators and potently inhibit melanoma-specific T-cell responses (173). This TIE-2/angiopoietin-2 signaling axis represents a novel tumor immune escape mechanism, and combining TIE-2 inhibitors with ICIs shows promise in overcoming MDSC-mediated suppression in melanoma (173).

In the translational I-RENE trial, HLA-DR- PD-L1^+^ MDSCs were reduced in metastatic clear cell renal carcinoma (RCC) responders upon anti-PD1 nivolumab treatment; however, patients with progressive disease and non-responders had an enriched MDSC signature (174). These studies highlight the importance of targeting MDSCs in resistant tumors that fail to respond to ICB.

## MDSC targeting with cancer vaccines and adoptive T-cell/NK-cell/macrophages

Recent preclinical studies have demonstrated that combining MDSC targeting with cancer vaccines and CAR-engineered immune cells can markedly enhance the antitumor efficacy in solid tumor models (175). Administration of an anti-Tumor mucin 1 (MUC1) vaccine in combination with tadalafil, a phosphodiesterase-5 (PDE5) inhibitor, significantly reduced both circulating and intratumoral MDSCs and Tregs while enhancing tumor-specific CD8 + T-cell responses in patients with head and neck squamous cell carcinoma (HNSCC) in a randomized clinical trial (169).

The efficacy of CAR-T cell therapy, especially third generation GD-2 specific CART cells, is hindered by high levels of peripheral PMN-MDSCs in neuroblastoma patients, illustrating the critical need to target MDSCs alongside adoptive cell therapy to maximize therapeutic outcomes (176). Preclinical evidence shows that pretreatment with the Bruton tyrosine kinase inhibitor ibrutinib can attenuate MDSC-driven immunosuppression of CART19 cell function *in vitro* (177). Moreover, when ibrutinib is combined with standard lymphodepleting chemotherapy, it further enhances the *in vivo* anti-tumor activity of CART19 cells by weakening the immunosuppressive TME (177).

A diverse set of small-molecule inhibitors provides additional combinatorial opportunities. For example, the PARP inhibitor olaparib disrupts the SDF-1/CXCR4 axis to impair MDSC recruitment, leading to superior tumor reduction when combined with EGFRvIII-targeted CAR-T cells (178). CAR-T cells engineered to co-express CXCR4 with CLDN18.2 block cancer-associated fibroblasts (CAF)-mediated recruitment of MDSCs, decrease infiltration of MDSCs, and increase CAR-T cell infiltration, ultimately enhancing the efficacy of CLDN18.2-positive PDAC models (179). The multi-targeted tyrosine kinase inhibitor lenvatinib lowers MDSC levels, impairs immunosuppression, boosts tumor-infiltrating T cell IFN-γ, and works synergistically with CAR-T therapy to reduce tumor burden (180). Anti-CD33 gemtuzumab ozogamicin depletes MDSCs and enhances the activity of anti-GD2-/mesothelin-/EGFRvIII-CAR T-cells (86). Activation of TLR3 by Poly I:C in conjunction with EGFRvIII-targeted CAR-T cells polarizes MDSCs toward tumoricidal M1 macrophages and produces durable synergistic anti-tumor responses (181).

Engineering CAR-T cells that engage MDSC-derived signals offers a dual-action approach that can robustly reprogram the TME (182). Dual-target CAR-T cells co-expressing mucin 1 (MUC1) and TRAIL-R2 (TR2.41BB) have improved efficacy by simultaneously targeting TR2 expressing MDSCs and optimally activating T cells via two co-stimulatory domains (CD28 and 41BB), supporting T cell persistence and expansion in the TME (183).

Fibroblast activation protein (FAP) is abundantly expressed by activated cancer-associated fibroblasts (CAFs) in pancreatic ductal adenocarcinoma, marking the desmoplastic stroma that impedes effective immunotherapy (184). In immunocompetent PDAC mouse models, sequential infusion of FAP-targeted CAR-T cells selectively eradicated FAP^+^ CAFs, leading to a pronounced drop in intratumoral CXCL12, which in turn disrupted MDSC recruitment and accumulation (185). This stromal remodeling creates a more permissive microenvironment for subsequent CLDN18.2-specific CAR-T cells, enhancing their infiltration, persistence, and antitumor activity at the tumor site (185).

In the CAR-NK landscape, NKG2D.ζ-engineered NK cells exploit high NKG2D ligand expression on MDSCs to selectively eliminate these suppressors, reprogram the cytokine milieu toward IFN-γ and TNF-α dominance, and rescue the efficacy of GD2-CAR-T cells in neuroblastoma xenografts (186).

Emerging CAR-macrophage (CAR-M) platforms, exemplified by anti-HER2 CAR-Ms, not only mediate the direct phagocytosis of tumor cells but also potentiate antigen presentation and pro-inflammatory cytokine release (187). Although clinical combinations with MDSC checkpoint inhibitors remain under exploration, theoretical models predict that blocking MDSC-mediated IL-10/TGF-β signaling will sustain CAR-M M1 polarization and reinforce durable antitumor immunity (187). CT-0508, a CAR-M platform, is currently undergoing phase 1 trials (NCT04660929) for HER2^+^ solid tumor malignancies (187).

### Applications of artificial intelligence in MDSC research

Artificial intelligence (AI), integrated with machine learning (ML) and deep learning (DL) approaches have shown remarkable potential in several areas of cancer research and clinical oncology, ranging from the early cancer detection and accurate diagnosis of malignancies to the development of personalized treatment designs informed by genomic, transcriptomic, proteomic, and metabolomic data (188). AI-driven systems are accelerating the discovery and validation of novel cancer biomarkers, enabling real-time assessment of tumor heterogeneity and dynamic monitoring of treatment response (189). ML and DL algorithms facilitate the identification of molecular targets and optimization of drug discovery pipelines (188). In the clinical setting, AI-based predictive models are increasingly being used to predict patient prognosis, immunotherapy outcomes and support precision oncology by integrating multi-omics and imaging data (188). In the context of MDSCs, Cchek™ liquid biopsy technology platform employs AI trained on flow cytometric immunophenotyping of MDSC subsets and lymphoid populations to accurately detect and distinguish early stages of malignant transformation (190). This approach facilitates early detection and diagnosis of multiple cancer types, including prostate, lung, breast and colon (190). The modelling of the predictive responseScore using graph neural networks (GNNs) to predict immunotherapy response and prognosis in bladder cancer could similarly be extended to the study of MDSCs (191). Such an approach may enable the prediction of patients exhibiting MDSC-driven resistance phenotypes. In addition, applying GNN models to single-cell transcriptomes of MDSCs could reveal distinct immunosuppressive and regulatory subpopulations, predict treatment response based on pathway activity, and uncover unique signaling networks associated with treatment resistance. Deep-learning algorithms currently used to quantify tumor-infiltrating lymphocytes (TILs) in melanoma can likewise be adapted to assess tumor-infiltrating MDSCs in solid malignancies from digital histopathological images (192). This approach could enable patient stratification based on the degree of MDSC infiltration and guide the selection of MDSC-targeted immunotherapeutic strategies. AI driven tools like LLM-scCurator that filter biological noise and extract relevant markers from single cell data could facilitate precise characterization of heterogenous MDSC subsets (193). Multimodal artificial intelligence (MMAI) frameworks, such as the deep learning-based DeepClinMed-PGM model, integrate histopathological imaging, genomic, transcriptomic, and metabolic data to predict disease-free survival in cancer patients (194). MMAI models could potentially identify patients with high predicted MDSC signatures who could benefit from MDSC targeted therapies.

## Challenges and future directions

Despite considerable progress in elucidating the immunosuppressive and tumor-promoting roles of MDSCs in the TME, several key challenges must be addressed in order to translate these insights into effective therapies. MDSCs exhibit profound subset-specific heterogeneity across both tumor types and individual patients, with variable phenotypic markers, immunosuppressive mechanisms, and metabolic reprogramming depending on the MDSC subsets, tissue type niche, complicating their identification and selective targeting. Hence, subset-specific analyses are crucial for refining therapeutic strategies aimed at MDSC depletion or functional inhibition. Global depletion of MDSCs risks unwanted immunotoxicity or compensatory expansion of therapy-resistant subsets. Conversely, targeting subset-specific markers (e.g., TIE-2 in M-MDSCs, CD100d in PMN-MDSCs) or metabolic dependencies (e.g., iNOS in M-MDSCs, NOX2 in PMN-MDSCs) could allow for precise modulation of immunosuppressive networks without compromising essential myeloid cell functions (**Table 3**). Further studies should therefore emphasize multi-parametric flow cytometry, single cell RNA sequencing, and metabolic profiling to clarify temporal evolution and functional plasticity of MDSC subsets within distinct microenvironments.

**Table 3:** Subset-specific metabolic dependencies and therapeutic approaches in MDSCs.

Feature	M-MDSC	PMN-MDSC	Therapeutic approach
Aerobic glycolysis	High; relies on mTOR/HIF-1α-mediated glycolysis for energy and suppressive function in hypoxic TME	Moderate; uses glycolysis to prevent ROS-mediated apoptosis, upregulates GLUT3 for glucose uptake under stress	MCT1/4 inhibitors to cause lactic acidosis (e.g., AZD3965, AZD0095); mTOR inhibitors (e.g., AZD2014), HIF-1α inhibitors (e.g., LW-6) or glycolysis pathway inhibitors (e.g., 2-DG, DCA) to impair glycolysis
OXPHOS	Low, shifts to glycolysis under hypoxia; less reliant compared to PMN-MDSCs	Moderate; supports energetic needs alongside glycolysis, regulated by AMPK	Complex 1 inhibitors (e.g., IACS-010759) to disrupt OXPHOS, AMPK activators (e.g., metformin) to hinder suppressive potential
FAO	High; PPARγ-mediated FAO for ATP and suppressive mediators; lipid uptake via CD36/FATP2 in lipid-rich TME	Moderate to High; FATP2 regulators AA and PGE2 for ROS production	FAO inhibitors (e.g., Etomoxir for CPT1A) to reduce ARG1/NO/ROS; FATP2 and EP4 inhibitors (e.g., Lipofermata, MF-766) to decrease lipid accumulation and PGE2 synthesis.
ROS production	Moderate; contributes to suppression but less dominant than in PMN-MDSCs, regulated by iNOS and STAT1	High, primary mechanism via NOX2, producing superoxide that forms peroxynitrite with NO	NOX2 inhibitors (histamine dihydrochloride) for reducing ROS production
NO production	High; produced by iNOS, inhibits T cell proliferation and induces apoptosis,	Low; reacts with ROS to form peroxynitrite, but less emphasis on direct NO production	iNOS inhibitors (L-NMMA) to reduce NO and restore T-cell activation, ATRA to lower iNOS expression

OXPHOS, Oxidative Phosphorylation; FAO, Fatty Acid Oxidation; PPARγ, Peroxisome Proliferator-Activated Receptor gamma; GLUT3, Glucose Transporter 3; AMPK, AMP-Activated Protein Kinase; 2-DG, 2-Deoxy-D-Glucose; DCA, Dichloroacetate; CPT1A, Carnitine Palmitoyltransferase 1A; AA, Arachidonic Acid; PGE2, Prostaglandin E2; EP4, Prostaglandin E2 receptor 4; NOX2, NADPH Oxidase 2; iNOS, Inducible Nitric Oxide Synthase; L-NMMA, N-monomethyl-L-arginine; ATRA, All Trans Retinoic Acid.

Within the evolving TME, MDSCs are capable of shifting their phenotypes in response to metabolic cues from the surrounding tumor or stromal cells (hypoxia, lactate) and resistance mechanisms following standard chemotherapies or immunotherapies, further hindering consistent phenotypic identification and selective targeting. Non-specific broad-range inhibitors targeting survival, recruitment, or differentiation of MDSCs risk collateral damage to essential myeloid populations (e.g., neutrophils and macrophages), potentially leading to neutropenia or impaired wound healing. To overcome these challenges, next-generation approaches are being developed to confine action to the tumor niche and to spare healthy myeloid compartments. For instance, antibody constructs that bridge MDSCs to cytotoxic T-cells only in the presence of tumor antigens (e.g., CD123 × CD3 BiTEs) promise minimal off-target myeloid depletion (195). Preclinical data suggest that CAR-T cells co-expressing receptors for MDSC‐derived signals (e.g., TR2.4-1BB) outperform unmodified CAR-T cells in solid models, but clinical translation is pending (183).

The lack of reliable blood or tissue markers to evaluate MDSC burden or functional state is a major barrier to patient selection and personalization of therapy. Although functional gene-expression signatures (such as S100A8/A9 and LOX‐1) are promising, robust prospective validation is lacking (196). Dynamic assays to monitor circulating MDSCs and their suppressive activity, along with the integration of advanced profiling technologies such as single-cell RNA sequencing, proteomics, spatial transcriptomics in patient biopsies, and imaging mass cytometry to spatially identify tumor-infiltrating MDSC signatures and immunosuppressive interactions within TME could allow precise stratification of patients and enable real-time adaptation of therapeutic regimens (197).

Efforts to enhance MDSC-targeted therapy specificity have focused on minimizing off-target effects and reducing systemic toxicities. Context-activated checkpoint inhibitors such as the novel pH-sensitive linker-based co-delivery of anti-PD-1 and Midkine-siRNA that unveil inhibitory domains only in the acidic TME potentially sharpen spatial precision to neutralize MDSCs (198). Protease-sensitive pro-antibodies whose Fc or Fab regions are masked until exposure to tumor-specific proteases could also restrict MDSC blockade to the malignant niche, reducing systemic toxicity (199). Concurrently, controlled delivery vehicles such as lipid or polymer-based nanoparticles and exosome-mimetic vesicles are being engineered to deliver metabolic modulators directly to intratumoral MDSCs, thereby enhancing the local potency while minimizing systemic toxicity (200).

Single-pathway blockade of MDSCs can trigger compensatory immunosuppressive circuits, necessitating multitarget regimens for durable responses (201). Combining MDSC inhibitors with immune checkpoint blockade and adoptive cellular therapies (such as CAR-T or CAR-M) is promising, but requires careful pharmacodynamic and scheduling optimization to avoid antagonism and maximize T cell priming (5). This intensified immune activation heightens the risk spectrum of adverse immune-related events, mandating routine immune monitoring and standardized mitigation protocols in clinical trials (202). Additionally, the complex crosstalk of MDSC subsets with Tregs, TAMs, and TANs reinforces the need for multi-targeted suppression of immunosuppressive and tumor-supporting networks within the TME.

The translation of advanced MDSC-targeting modalities will depend on embedding adaptive, biomarker-guided trial designs testing multiple MDSC-targeted approaches, subset-specific MDSC analyses and real-time monitoring (e.g., PET imaging of surface level and functional MDSC signatures) to stratify patients and personalize regimens. Integration of single-cell multi-omics profiling, artificial intelligence, deep learning platforms, and longitudinal monitoring will enable early identification of non-responders, predict prognosis in responders displaying MDSC signatures, and validate the safety and long-term efficacy of immune-metabolic targeting of MDSCs in solid and hematological malignancies.

## Conclusions

MDSCs play a central role in shaping the immunosuppressive TME and represent a major barrier to the success of current immunotherapy. Advances over the past decade have greatly expanded our understanding of their ontogeny, metabolic adaptations, and functional interactions with other immune and stromal cell populations. These insights have catalyzed the development of multiple therapeutic strategies, including metabolic modulation, direct depletion, and inhibition of recruitment and signaling, and promotion of differentiation, many of which have shown strong preclinical efficacy and are advancing into clinical trials. However, significant challenges remain, including the heterogeneity and plasticity of MDSC populations, lack of standardized biomarkers, and need for precise, selective interventions that minimize collateral effects on other immune compartments. The rational integration of MDSC-targeting approaches with immune checkpoint blockade, cancer vaccines, and adoptive cell therapies holds particular promise for overcoming resistance and achieving durable tumor control. A combination of mechanistic insights from immunometabolism and spatial profiling, coupled with innovative therapeutic design and biomarker-guided clinical translation, will be essential to fully realize the potential of MDSC-targeted interventions. By dismantling these key suppressive networks, MDSC-focused strategies have the potential to broaden and deepen the responses to cancer immunotherapy and improve patient outcomes across a wide spectrum of malignancies.

## Glossary

GM-CSF: Granulocyte–Macrophage Colony-Stimulating Factor
M-CSF: Macrophage Colony-Stimulating Factor
G-CSF: Granulocyte Colony-Stimulating Factor
SCF: Stem Cell Factor
IL-6: Interleukin-6
IL-10: Interleukin-10
IL-1β: Interleukin-1β
VEGF: Vascular Endothelial Growth Factor
HIF1a: Hypoxia Inducible Factor 1a
S100A8: S100 calcium binding protein 8
S100A9: S100 calcium binding protein 9
TNF-α: Tumor Necrosis Factor-a
JAK1/2: Janus Kinase ½
STAT: Signal Transducer and Activator of Transcription
LAP: Leukocyte Alkaline Phosphatase
NF-kB: Nuclear Factor-kappa B
PGE2: Prostaglandin E2
IFN-g: Interferon-g
IRF: Interferon Regulatory Factor
CXCL: C-X-C motif Chemokine Ligand
CCL: C-C motif Chemokine Ligand
HLA: Human Leukocyte Antigen
JAM1: Junctional Adhesion Molecule 1
TREM2: Triggering Receptor Expressed on Myeloid Cells 2
SPARC: Secreted Protein, Acidic and Rich in Cysteine
AA: Arachidonic Acid
FATP2: Fatty Acid Transport Protein 2
LOX1: Lectin-like oxidized low-density lipoprotein receptor-1
CD: Cluster of Differentiation
LXR: Liver X Receptor
TRAIL-R2: TNF-related apoptosis-inducing ligand receptor 2
TAM-RTK: TYRO3, AXL and MERTK family of receptor tyrosine kinases
EGFR: Epidermal Growth Factor Receptor
BTK: Bruton’s Tyrosine Kinase
CCR5: Chemokine Receptor type 5
MEK1/2: Mitogen-Activated Protein Kinase ½
MyD88: Myeloid Differentiation Factor 88
Cys: Cystine
Trp: Tryptophan
L-Kyn: L-kynurenine
GLS: Glutaminase
GLUT1: Glucose Transporter 1
GLUT3: Glucose Transporter 3
L-NMMA: N-monomethyl-L-arginine
FAO: Fatty Acid Oxidation
OXPHOS: Oxidative Phosphorylation
PDH: Pyruvate Dehydrogenase
L-Arg: L-Arginine
Gln: Glutamine
TCA cycle: Tricarboxylic Acid cycle
IDO1: Indoleamine 2,3 –dioxygenase
LAT1: L-type Amino Acid transporter 1
CAT2B: Cationic Amino Acid Transporter 2B
MIF2: Macrophage migration Inhibitory Factor 2
CPT1A: Carnitine Palmitoyltransferase 1A
NOX2: NADPH Oxidase 2
PPARγ: Peroxisome Proliferator-Activated Receptor gamma
AMPK: AMP-Activated Protein Kinase
2-DG: 2-Deoxy-D-Glucose
DCA: Dichloroacetate
CPT1A: Carnitine Palmitoyltransferase 1A
EP4: Prostaglandin E2 receptor 4
iNOS: Inducible Nitric Oxide Synthase
TIE-2: Tyrosine kinase with Ig and epidermal growth factor (EGF) homology domain 2
VDR: Vitamin D Receptor
FAP: Fibroblast activation protein
CAF: Cancer-associated fibroblasts
Tregs: Regulatory T cell
TAM: Tumor Associated Macrophage
TAN: Tumor Associated Neutrophil
PET: Position Electron Tomography
PDE5: Phosphodiesterase-5
PDAC: Pancreatic Adenocarcinoma
AML: Acute Myeloid Leukemia
MDS: Myelodysplastic syndrome
NSCLC: Non-Small Cell Lung Cancer
SCLC: Small Cell Lung Cancer
CLL: Chronic Lymphoblastic Leukemia
SLL: Small Lymphoblastic Leukemia
RCC: Renal Cell Carcinoma
CRC: Colorectal Cancer
LV: Leucovorin
5-FU: 5-Fluorouracil
NK cell: natural killer cell
DC: Dendritic cell

## References

[1] Wen H Zheng S Zhu X Wang L Chen D . Characteristics of the tumor microenvironment and potential immunotherapy strategies in renal cell carcinoma. Front Immunol. (2025) 16:1643533. doi: 10.3389/fimmu.2025.1643533, PMID: 40963620 PMC12436424

[2] Ma W Baran N . Expanding horizons in esophageal squamous cell carcinoma: The promise of induction chemoimmunotherapy with radiotherapy. World J Clin Oncol. (2025) 16:104959. doi: 10.5306/wjco.v16.i7.104959, PMID: 40741200 PMC12304926

[3] Abdin SM Paasch D Morgan M Lachmann N . CARs and beyond: Tailoring macrophage-based cell therapeutics to combat solid Malignancies. J Immunother Cancer. (2021) 9:e002741. doi: 10.1136/jitc-2021-002741, PMID: 34462325 PMC8407221

[4] Yaseen MM Abuharfeil NM Darmani H Daoud A . Recent advances in myeloid-derived suppressor cell biology. Front Med. (2021) 15:232–51. doi: 10.1007/s11684-020-0797-2, PMID: 32876877

[5] Ibrahim A Abdalsalam N Liang Z Kashaf Tariq H Li R O. Afolabi L . MDSC checkpoint blockade therapy: a new breakthrough point for overcoming immunosuppression in cancer immunotherapy. Cancer Gene Ther. (2025) 32:371–92. doi: 10.1038/s41417-025-00886-9, PMID: 40140724 PMC11976280

[6] Wang H Zhou F Qin W Yang Y Li X Liu R . Metabolic regulation of myeloid-derived suppressor cells in tumor immune microenvironment: Targets and therapeutic strategies. Theranostics. (2025) 15:2159–84. doi: 10.7150/thno.105276, PMID: 39990210 PMC11840731

[7] He S Zheng L Qi C . Myeloid-derived suppressor cells (MDSCs) in the tumor microenvironment and their targeting in cancer therapy. Mol Cancer. (2025) 24:5. doi: 10.1186/s12943-024-02208-3, PMID: 39780248 PMC11707952

[8] Kumar D Da Silva VC Chaves NL . Myeloid-derived suppressor cells as targets of emerging therapies and nanotherapies (review). Med Int (Lond). (2024) 4:46. doi: 10.3892/mi.2024.170, PMID: 38983795 PMC11228699

[9] Cassetta L Bruderek K Skrzeczynska-Moncznik J Osiecka O Hu X Rundgren IM . Differential expansion of circulating human MDSC subsets in patients with cancer, infection and inflammation. J Immunother Cancer. (2020) 8:e001223. doi: 10.1136/jitc-2020-001223, PMID: 32907925 PMC7481096

[10] Kumar V Patel S Tcyganov E Gabrilovich DI . The Nature of myeloid-derived suppressor cells in the tumor microenvironment. Trends Immunol. (2016) 37:208–20. doi: 10.1016/j.it.2016.01.004, PMID: 26858199 PMC4775398

[11] Bronte V Brandau S Chen SH Colombo MP Frey AB Greten TF . Recommendations for myeloid-derived suppressor cell nomenclature and characterization standards. Nat Commun. (2016) 7:12150. doi: 10.1038/ncomms12150, PMID: 27381735 PMC4935811

[12] Kohada Y Kuromoto A Takeda K Iwamura H Atobe Y Ito J . Circulating PMN-MDSC levels positively correlates with a poor prognosis in patients with metastatic hormone-sensitive prostate cancer. Front Urol. (2022) 2. doi: 10.3389/fruro.2022.967480, PMID: 41728591

[13] Saylor J Ma Z Goodridge HS Huang F Cress AE Pandol SJ . Spatial mapping of myeloid cells and macrophages by multiplexed tissue staining. Front Immunol. (2018) 9:2925. doi: 10.3389/fimmu.2018.02925, PMID: 30619287 PMC6302234

[14] Wang C Zheng X Zhang J Jiang X Wang J Li Y . CD300ld on neutrophils is required for tumour-driven immune suppression. Nature. (2023) 621:830–9. doi: 10.1038/s41586-023-06511-9, PMID: 37674079

[15] Khan ANH Emmons TR Wong JT Alqassim E Singel KL Mark J . Quantification of early-stage myeloid-derived suppressor cells in cancer requires excluding basophils. Cancer Immunol Res. (2020) 8:819–28. doi: 10.1158/2326-6066.CIR-19-0556, PMID: 32238380 PMC7269807

[16] De Cicco P Ercolano G Ianaro A . The New Era of cancer immunotherapy: Targeting myeloid-derived suppressor cells to overcome immune evasion. Front Immunol. (2020) 11. doi: 10.3389/fimmu.2020.01680, PMID: 32849585 PMC7406792

[17] Karadima E Chavakis T Alexaki VI . Arginine metabolism in myeloid cells in health and disease. Semin Immunopathol. (2025) 47:11. doi: 10.1007/s00281-025-01038-9, PMID: 39863828 PMC11762783

[18] Seo S-K Kwon B . Immune regulation through tryptophan metabolism. Exp Mol Med. (2023) 55:1371–9. doi: 10.1038/s12276-023-01028-7, PMID: 37394584 PMC10394086

[19] Baumann T Dunkel A Schmid C Schmitt S Hiltensperger M Lohr K . Regulatory myeloid cells paralyze T cells through cell-cell transfer of the metabolite methylglyoxal. Nat Immunol. (2020) 21:555–66. doi: 10.1038/s41590-020-0666-9, PMID: 32327756

[20] Nagatani Y Funakoshi Y Suto H Imamura Y Toyoda M Kiyota N . Immunosuppressive effects and mechanisms of three myeloid-derived suppressor cells subsets including monocytic-myeloid-derived suppressor cells, granulocytic-myeloid-derived suppressor cells, and immature-myeloid-derived suppressor cells. J Cancer Res Ther. (2021) 17:1093–100. doi: 10.4103/jcrt.JCRT_1222_20, PMID: 34528569

[21] Li BH Garstka MA Li ZF . Chemokines and their receptors promoting the recruitment of myeloid-derived suppressor cells into the tumor. Mol Immunol. (2020) 117:201–15. doi: 10.1016/j.molimm.2019.11.014, PMID: 31835202

[22] Deng Y Yang J Luo F Qian J Liu R Zhang D . mTOR-mediated glycolysis contributes to the enhanced suppressive function of murine tumor-infiltrating monocytic myeloid-derived suppressor cells. Cancer Immunol Immunother. (2018) 67:1355–64. doi: 10.1007/s00262-018-2177-1, PMID: 29968153 PMC11028128

[23] Yue J Li J Ma J Zhai Y Shen L Zhang W . Myeloid-derived suppressor cells inhibit natural killer cells in myelodysplastic syndromes through the TIGIT/CD155 pathway. Hematology. (2023) 28:2166333. doi: 10.1080/16078454.2023.2166333, PMID: 36651499

[24] Limagne E Richard C Thibaudin M Fumet JD Truntzer C Lagrange A . Tim-3/galectin-9 pathway and mMDSC control primary and secondary resistances to PD-1 blockade in lung cancer patients. Oncoimmunology. (2019) 8:e1564505. doi: 10.1080/2162402X.2018.1564505, PMID: 30906658 PMC6422400

[25] Wang Y Schafer CC Hough KP Tousif S Duncan SR Kearney JF . Myeloid-derived suppressor cells impair B cell responses in lung cancer through IL-7 and STAT5. J Immunol. (2018) 201:278–95. doi: 10.4049/jimmunol.1701069, PMID: 29752311 PMC6008229

[26] Ugolini A Tyurin VA Tyurina YY Tcyganov EN Donthireddy L Kagan VE . Polymorphonuclear myeloid-derived suppressor cells limit antigen cross-presentation by dendritic cells in cancer. JCI Insight. (2020) 5:e138581. doi: 10.1172/jci.insight.138581, PMID: 32584791 PMC7455061

[27] Xia C Yin S To KKW Fu L . CD39/CD73/A2AR pathway and cancer immunotherapy. Mol Cancer. (2023) 22:44. doi: 10.1186/s12943-023-01733-x, PMID: 36859386 PMC9979453

[28] Mandula JK Rodriguez PC . Tumor-related stress regulates functional plasticity of MDSCs. Cell Immunol. (2021) 363:104312. doi: 10.1016/j.cellimm.2021.104312, PMID: 33652258 PMC8026602

[29] Suri C Pande B Suhasini Sahithi L Swarnkar S Khelkar T Verma HK . Metabolic crossroads: Unravelling immune cell dynamics in gastrointestinal cancer drug resistance. Cancer Drug Resist. (2025) 8:7. doi: 10.20517/cdr.2024.164, PMID: 40051496 PMC11883236

[30] Goffaux G Hammami I Jolicoeur M . A dynamic metabolic flux analysis of myeloid-derived suppressor cells confirms immunosuppression-related metabolic plasticity. Sci Rep. (2017) 7:9850. doi: 10.1038/s41598-017-10464-1, PMID: 28852166 PMC5575287

[31] Fu C Fu Z Jiang C Xia C Zhang Y Gu X . CD205+ polymorphonuclear myeloid-derived suppressor cells suppress antitumor immunity by overexpressing GLUT3. Cancer Sci. (2021) 112:1011–25. doi: 10.1111/cas.14783, PMID: 33368883 PMC7935791

[32] Meng G Li B Chen A Zheng M Xu T Zhang H . Targeting aerobic glycolysis by dichloroacetate improves Newcastle disease virus-mediated viro-immunotherapy in hepatocellular carcinoma. Br J Cancer. (2020) 122:111–20. doi: 10.1038/s41416-019-0639-7, PMID: 31819179 PMC6964686

[33] Jia L Gao Y Zhou T Zhao XL Hu HY Chen DW . Enhanced response to PD-L1 silencing by modulation of TME via balancing glucose metabolism and robust co-delivery of siRNA/resveratrol with dual-responsive polyplexes. Biomaterials. (2021) 271:120711. doi: 10.1016/j.biomaterials.2021.120711, PMID: 33592352

[34] Xiong D Yin Z Huang M Wang Y Hardy M Kalyanaraman B . Mitochondria-targeted atovaquone promotes anti-lung cancer immunity by reshaping tumor microenvironment and enhancing energy metabolism of anti-tumor immune cells. Cancer Commun (Lond). (2024) 44:448–52. doi: 10.1002/cac2.12500, PMID: 37930151 PMC10958673

[35] Baran N Patel S Lodi A Ortiz JE Dhungana Y Collins M . Accumulation of intracellular L-lactate and irreversible disruption of mitochondrial membrane potential upon dual inhibition of Oxphos and lactate transporter MCT-1 induce synthetic lethality in T-ALL Via mitochondrial exhaustion. Blood. (2021) 138:680–. doi: 10.1182/blood-2021-152845, PMID: 41496790

[36] Firmanty P Chomczyk M Dash S Konopleva M Baran N . Feasibility and safety of targeting mitochondria function and metabolism in acute myeloid leukemia. Curr Pharmacol Rep. (2024) 10:388–404. doi: 10.1007/s40495-024-00378-8, PMID: 40756330 PMC12314886

[37] Sandanayaka V Sharma S Bowman N Duffy J Wijerathna S Yu M . Cellular transporters as novel metabolic immune checkpoints: NGY-091, a small molecule dual MCT1/4 inhibitor for immuno oncology. Eur J Cancer. (2022) 174:S127. doi: 10.1016/S0959-8049(22)01137-6, PMID: 41698777

[38] Trillo-Tinoco J Sierra RA Mohamed E Cao Y de Mingo-Pulido Á Gilvary DL . AMPK Alpha-1 intrinsically regulates the function and differentiation of tumor myeloid-derived suppressor cells. Cancer Res. (2019) 79:5034–47. doi: 10.1158/0008-5472.CAN-19-0880, PMID: 31409640 PMC6774829

[39] Li L Wang L Li J Fan Z Yang L Zhang Z . Metformin-induced reduction of CD39 and CD73 blocks myeloid-derived suppressor cell activity in patients with ovarian cancer. Cancer Res. (2018) 78:1779–91. doi: 10.1158/0008-5472.CAN-17-2460, PMID: 29374065 PMC5882589

[40] Qin G Lian J Huang L Zhao Q Liu S Zhang Z . Metformin blocks myeloid-derived suppressor cell accumulation through AMPK-DACH1-CXCL1 axis. Oncoimmunology. (2018) 7:e1442167. doi: 10.1080/2162402X.2018.1442167, PMID: 29900050 PMC5993496

[41] Huang K Han Y Chen Y Shen H Zeng S Cai C . Tumor metabolic regulators: Key drivers of metabolic reprogramming and the promising targets in cancer therapy. Mol Cancer. (2025) 24:7. doi: 10.1186/s12943-024-02205-6, PMID: 39789606 PMC11716519

[42] Sosnowska A Chlebowska-Tuz J Matryba P Pilch Z Greig A Wolny A . Inhibition of arginase modulates T-cell response in the tumor microenvironment of lung carcinoma. Oncoimmunology. (2021) 10:1956143. doi: 10.1080/2162402X.2021.1956143, PMID: 34367736 PMC8312619

[43] Steggerda SM Bennett MK Chen J Emberley E Huang T Janes JR . Inhibition of arginase by CB-1158 blocks myeloid cell-mediated immune suppression in the tumor microenvironment. J Immunother Cancer. (2017) 5:101. doi: 10.1186/s40425-017-0308-4, PMID: 29254508 PMC5735564

[44] Grzybowski MM Stańczak PS Pomper P Błaszczyk R Borek B Gzik A . OATD-02 validates the benefits of pharmacological inhibition of arginase 1 and 2 in cancer. Cancers (Basel). (2022) 14:3967. doi: 10.3390/cancers14163967, PMID: 36010962 PMC9406419

[45] Borek B Nowicka J Gzik A Dziegielewski M Jedrzejczak K Brzezinska J . Arginase 1/2 inhibitor OATD-02: From discovery to first-in-man setup in cancer immunotherapy. Mol Cancer Ther. (2023) 22:807–17. doi: 10.1158/1535-7163.MCT-22-0721, PMID: 36939275

[46] Poon E Mullins S Watkins A Williams GS Koopmann JO Di Genova G . The MEK inhibitor selumetinib complements CTLA-4 blockade by reprogramming the tumor immune microenvironment. J Immunother Cancer. (2017) 5:63. doi: 10.1186/s40425-017-0268-8, PMID: 28807001 PMC5557252

[47] Miret JJ Kirschmeier P Koyama S Zhu M Li YY Naito Y . Suppression of Myeloid Cell arginase Activity leads to Therapeutic Response in a NSCLC Mouse Model by Activating anti-tumor Immunity. J Immunother Cancer. (2019) 7:32. doi: 10.1186/s40425-019-0504-5, PMID: 30728077 PMC6366094

[48] Mondanelli G Bianchi R Pallotta MT Orabona C Albini E Iacono A . A relay pathway between arginine and tryptophan metabolism confers immunosuppressive properties on dendritic cells. Immunity. (2017) 46:233–44. doi: 10.1016/j.immuni.2017.01.005, PMID: 28214225 PMC5337620

[49] Bian Z Abdelaal AM Shi L Liang H Xiong L Kidder K . Arginase-1 is neither constitutively expressed in nor required for myeloid-derived suppressor cell-mediated inhibition of T-cell proliferation. Eur J Immunol. (2018) 48:1046–58. doi: 10.1002/eji.201747355, PMID: 29488625 PMC5997508

[50] Dey S Mondal A DuHadaway JB Sutanto-Ward E Laury-Kleintop LD Thomas S . IDO1 signaling through GCN2 in a subpopulation of Gr-1+ cells shifts the IFNγ/IL6 balance to promote neovascularization. Cancer Immunol Res. (2021) 9:514–28. doi: 10.1158/2326-6066.CIR-20-0226, PMID: 33622713

[51] Tousif S Wang Y Jackson J Hough KP Strenkowski JG Athar M . Indoleamine 2, 3-dioxygenase promotes aryl hydrocarbon receptor-dependent differentiation of regulatory B cells in lung cancer. Front Immunol. (2021) 12:747780. doi: 10.3389/fimmu.2021.747780, PMID: 34867973 PMC8640488

[52] Li A Barsoumian HB Schoenhals JE Cushman TR Caetano MS Wang X . Indoleamine 2,3-dioxygenase 1 inhibition targets anti-PD1-resistant lung tumors by blocking myeloid-derived suppressor cells. Cancer Lett. (2018) 431:54–63. doi: 10.1016/j.canlet.2018.05.005, PMID: 29746927 PMC6027590

[53] Liang H Li T Fang X Xing Z Zhang S Shi L . IDO1/TDO dual inhibitor RY103 targets Kyn-AhR pathway and exhibits preclinical efficacy on pancreatic cancer. Cancer Lett. (2021) 522:32–43. doi: 10.1016/j.canlet.2021.09.012, PMID: 34520819

[54] Xing Z Li X He ZNT Fang X Liang H Kuang C . IDO1 inhibitor RY103 suppresses Trp-GCN2-mediated angiogenesis and counters immunosuppression in glioblastoma. Pharmaceutics. (2024) 16:870. doi: 10.3390/pharmaceutics16070870, PMID: 39065567 PMC11279595

[55] Kenski JCN Huang X Vredevoogd DW de Bruijn B Traets JJH Ibáñez-Molero S . An adverse tumor-protective effect of IDO1 inhibition. Cell Rep Med. (2023) 4:100941. doi: 10.1016/j.xcrm.2023.100941, PMID: 36812891 PMC9975322

[56] Wang Z Li W Jiang Y Tran TB Cordova LE Chung J . Sphingomyelin-derived nanovesicles for the delivery of the IDO1 inhibitor epacadostat enhance metastatic and post-surgical melanoma immunotherapy. Nat Commun. (2023) 14:7235. doi: 10.1038/s41467-023-43079-4, PMID: 37945606 PMC10636136

[57] Wu C Spector SA Theodoropoulos G Nguyen DJM Kim EY Garcia A . Dual inhibition of IDO1/TDO2 enhances anti-tumor immunity in platinum-resistant non-small cell lung cancer. Cancer Metab. (2023) 11:7. doi: 10.1186/s40170-023-00307-1, PMID: 37226257 PMC10207715

[58] Groth C Weber R Utikal J Umansky V . Depletion and maturation of myeloid-derived suppressor cells in murine cancer models. Methods Mol Biol. (2021) 2236:67–75. doi: 10.1007/978-1-0716-1060-2_7, PMID: 33237541

[59] Kumar MA Baba SK Khan IR Khan MS Husain FM Ahmad S . Glutamine metabolism: Molecular regulation, biological functions, and diseases. MedComm. (2025) 6:e70120. doi: 10.1002/mco2.70120, PMID: 40567251 PMC12188105

[60] Oh MH Sun IH Zhao L Leone RD Sun IM Xu W . Targeting glutamine metabolism enhances tumor-specific immunity by modulating suppressive myeloid cells. J Clin Invest. (2020) 130:3865–84. doi: 10.1172/JCI131859, PMID: 32324593 PMC7324212

[61] Daneshmandi S Choi JE Yan Q MacDonald CR Pandey M Goruganthu M . Myeloid-derived suppressor cell mitochondrial fitness governs chemotherapeutic efficacy in hematologic Malignancies. Nat Commun. (2024) 15:2803. doi: 10.1038/s41467-024-47096-9, PMID: 38555305 PMC10981707

[62] Lemberg KM Vornov JJ Rais R Slusher BS . We’re not “DON” yet: Optimal dosing and prodrug delivery of 6-diazo-5-oxo-L-norleucine. Mol Cancer Ther. (2018) 17:1824–32. doi: 10.1158/1535-7163.MCT-17-1148, PMID: 30181331 PMC6130910

[63] Huang M Xiong D Pan J Zhang Q Sei S Shoemaker RH . Targeting glutamine metabolism to enhance Immunoprevention of EGFR-driven lung cancer. Adv Sci (Weinh). (2022) 9:e2105885. doi: 10.1002/advs.202105885, PMID: 35861366 PMC9475521

[64] Yokoyama Y Estok TM Wild R . Sirpiglenastat (DRP-104) induces antitumor efficacy through direct, Broad antagonism of glutamine metabolism and stimulation of the innate and adaptive immune systems. Mol Cancer Ther. (2022) 21:1561–72. doi: 10.1158/1535-7163.MCT-22-0282, PMID: 35930753

[65] Rais R Lemberg KM Tenora L Arwood ML Pal A Alt J . Discovery of DRP-104, a tumor-targeted metabolic inhibitor prodrug. Sci Adv. (2022) 8:eabq5925. doi: 10.1126/sciadv.abq5925, PMID: 36383674 PMC9668306

[66] Gouda MA Voss MH Tawbi H Gordon M Tykodi SS Lam ET . A phase I/II study of the safety and efficacy of telaglenastat (CB-839) in combination with nivolumab in patients with metastatic melanoma, renal cell carcinoma, and non-small-cell lung cancer. ESMO Open. (2025) 10:104536. doi: 10.1016/j.esmoop.2025.104536, PMID: 40359708 PMC12141888

[67] Zhang N Ping W Rao K Zhang Z Huang R Zhu D . Biomimetic copper-doped polypyrrole nanoparticles induce glutamine metabolism inhibition to enhance breast cancer cuproptosis and immunotherapy. J Control Release. (2024) 371:204–15. doi: 10.1016/j.jconrel.2024.05.045, PMID: 38810704

[68] Guo H Li W Pan G Wang C Li D Liu N . The glutaminase inhibitor Compound 968 exhibits potent *in vitro* and *in vivo* anti-tumor effects in endometrial cancer. Anti-Cancer Agents Med Chem. (2023) 23:210–21. doi: 10.2174/1871520622666220513163341, PMID: 35570522

[69] Timofeeva N Ayres ML Baran N Santiago-O’Farrill JM Bildik G Lu Z . Preclinical investigations of the efficacy of the glutaminase inhibitor CB-839 alone and in combinations in chronic lymphocytic leukemia. Front Oncol. (2023) 13:1161254. doi: 10.3389/fonc.2023.1161254, PMID: 37228498 PMC10203524

[70] DiNardo CD Verma D Baran N Bhagat TD Skwarska A Lodi A . Glutaminase inhibition in combination with azacytidine in myelodysplastic syndromes: A phase 1b/2 clinical trial and correlative analyses. Nat Cancer. (2024) 5:1515–33. doi: 10.1038/s43018-024-00811-3, PMID: 39300320 PMC13318259

[71] Ruiu R Cossu C Iacoviello A Conti L Bolli E Ponzone L . Cystine/glutamate antiporter xCT deficiency reduces metastasis without impairing immune system function in breast cancer mouse models. J Exp Clin Cancer Res. (2023) 42:254. doi: 10.1186/s13046-023-02830-x, PMID: 37770957 PMC10540318

[72] Yan D Adeshakin AO Xu M Afolabi LO Zhang G Chen YH . Lipid metabolic pathways confer the immunosuppressive function of myeloid-derived suppressor cells in tumor. Front Immunol. (2019) 10:1399. doi: 10.3389/fimmu.2019.01399, PMID: 31275326 PMC6593140

[73] Perez VM Gabell J Behrens M Wase N DiRusso CC Black PN . Deletion of fatty acid transport protein 2 (FATP2) in the mouse liver changes the metabolic landscape by increasing the expression of PPARalpha-regulated genes. J Biol Chem. (2020) 295:5737–50. doi: 10.1074/jbc.RA120.012730, PMID: 32188695 PMC7186177

[74] Veglia F Tyurin VA Blasi M De Leo A Kossenkov AV Donthireddy L . Fatty acid transport protein 2 reprograms neutrophils in cancer. Nature. (2019) 569:73–8. doi: 10.1038/s41586-019-1118-2, PMID: 30996346 PMC6557120

[75] Adeshakin AO Liu W Adeshakin FO Afolabi LO Zhang M Zhang G . Regulation of ROS in myeloid-derived suppressor cells through targeting fatty acid transport protein 2 enhanced anti-PD-L1 tumor immunotherapy. Cell Immunol. (2021) 362:104286. doi: 10.1016/j.cellimm.2021.104286, PMID: 33524739

[76] Ching MM Reader J Fulton AM . Eicosanoids in cancer: Prostaglandin E2 receptor 4 in cancer therapeutics and immunotherapy. Front Pharmacol. (2020) 11:819. doi: 10.3389/fphar.2020.00819, PMID: 32547404 PMC7273839

[77] Mohammadpour H MacDonald CR McCarthy PL Abrams SI Repasky EA . beta2-adrenergic receptor signaling regulates metabolic pathways critical to myeloid-derived suppressor cell function within the TME. Cell Rep. (2021) 37:109883. doi: 10.1016/j.celrep.2021.109883, PMID: 34706232 PMC8601406

[78] Schuster C Akslen LA Straume O . beta2-adrenergic receptor expression in patients receiving bevacizumab therapy for metastatic melanoma. Cancer Med. (2023) 12:17891–900. doi: 10.1002/cam4.6424, PMID: 37551424 PMC10524038

[79] Brown DN Caffa I Cirmena G Piras D Garuti A Gallo M . Squalene epoxidase is a bona fide oncogene by amplification with clinical relevance in breast cancer. Sci Rep. (2016) 6:19435. doi: 10.1038/srep19435, PMID: 26777065 PMC4726025

[80] Zhu H Klement JD Lu C Redd PS Yang D Smith AD . Asah2 represses the p53–Hmox1 axis to protect myeloid-derived suppressor cells from ferroptosis. J Immunol. (2021) 206:1395–404. doi: 10.4049/jimmunol.2000500, PMID: 33547170 PMC7946776

[81] Yan D Wang J Sun H Zamani A Zhang H Chen W . TIPE2 specifies the functional polarization of myeloid-derived suppressor cells during tumorigenesis. J Exp Med. (2020) 217:e20182005. doi: 10.1084/jem.20182005, PMID: 31662347 PMC7041705

[82] Pan J Liang H Zhou L Lu W Huo B Liu R . SQLE-mediated squalene metabolism promotes tumor immune evasion in pancreatic cancer. Front Immunol. (2024) 15:1512981. doi: 10.3389/fimmu.2024.1512981, PMID: 39763673 PMC11701373

[83] Wen J Zhang X Wong CC Zhang Y Pan Y Zhou Y . Targeting squalene epoxidase restores anti-PD-1 efficacy in metabolic dysfunction-associated steatohepatitis-induced hepatocellular carcinoma. Gut. (2024) 73:2023–36. doi: 10.1136/gutjnl-2023-331117, PMID: 38744443 PMC11671884

[84] Wu J Hu W Yang W Long Y Chen K Li F . Knockdown of SQLE promotes CD8+ T cell infiltration in the tumor microenvironment. Cell Signal. (2024) 114:110983. doi: 10.1016/j.cellsig.2023.110983, PMID: 37993027

[85] Tariq HK Liang Z Rabiu L Ibrahim A Mohamady Farouk Abdalsalam N Li R . Blockade of TIPE2-mediated ferroptosis of myeloid-derived suppressor cells achieves the full potential of combinatory ferroptosis and anti-PD-L1 cancer immunotherapy. Cells. (2025) 14:108. doi: 10.3390/cells14020108, PMID: 39851538 PMC11763990

[86] Fultang L Panetti S Ng M Collins P Graef S Rizkalla N . MDSC targeting with gemtuzumab ozogamicin restores T cell immunity and immunotherapy against cancers. EBiomedicine. (2019) 47:235–46. doi: 10.1016/j.ebiom.2019.08.025, PMID: 31462392 PMC6796554

[87] Chin A Jiao R Allen KJH Li J Chen M Vusirikala M . Lintuzumab-Ac225, a CD33-directed antibody radiotherapy, targets AML in a mutation agnostic manner. Blood. (2023) 142:5750. doi: 10.1182/blood-2023-187498, PMID: 41496790

[88] Komrokji RS Carraway HE Germing U Wermke M Zeidan AM Fu E . A phase I/II multicenter, open-label, dose escalation and randomized trial of BI 836858 in patients with low- or intermediate-1-risk myelodysplastic syndrome. Haematologica. (2022) 107:2742–7. doi: 10.3324/haematol.2021.280500, PMID: 35734924 PMC9614517

[89] Jitschin R Saul D Braun M Tohumeken S Völkl S Kischel R . CD33/CD3-bispecific T-cell engaging (BiTE^®^) antibody construct targets monocytic AML myeloid-derived suppressor cells. J Immunother Cancer. (2018) 6:116. doi: 10.1186/s40425-018-0432-9, PMID: 30396365 PMC6217777

[90] Cheng P Chen X Dalton R Calescibetta A So T Gilvary D . Immunodepletion of MDSC by AMV564, a novel bivalent, bispecific CD33/CD3 T cell engager, ex vivo in MDS and melanoma. Mol Ther. (2022) 30:2315–26. doi: 10.1016/j.ymthe.2022.02.005, PMID: 35150889 PMC9171150

[91] Erica DW Daniel JW Daniel AV Rose W Dixie L JoAnn K . GTB-3550 TriKE™ for the treatment of high-risk myelodysplastic syndromes (MDS) and refractory/relapsed acute myeloid leukemia (AML) safely drives natural killer (NK) cell proliferation at initial dose cohorts. Blood. (2020) 136:7–8. doi: 10.1182/blood-2020-136398, PMID: 41496790

[92] Felices M Lenvik TR Kodal B Lenvik AJ Hinderlie P Bendzick LE . Potent cytolytic activity and specific IL15 delivery in a second-generation trispecific killer engager. Cancer Immunol Res. (2020) 8:1139–49. doi: 10.1158/2326-6066.CIR-19-0837, PMID: 32661096 PMC7484162

[93] Tang Y Zhou C Li Q Cheng X Huang T Li F . Targeting depletion of myeloid-derived suppressor cells potentiates PD-L1 blockade efficacy in gastric and colon cancers. Oncoimmunology. (2022) 11:2131084. doi: 10.1080/2162402X.2022.2131084, PMID: 36268178 PMC9578486

[94] Jin R Liu L Xing Y Meng T Ma L Pei J . Dual mechanisms of novel CD73-targeted antibody and antibody-drug conjugate in inhibiting lung tumor growth and promoting antitumor immune-effector function. Mol Cancer Ther. (2020) 19:2340–52. doi: 10.1158/1535-7163.MCT-20-0076, PMID: 32943546

[95] Choueiry F Torok M Shakya R Agrawal K Deems A Benner B . CD200 promotes immunosuppression in the pancreatic tumor microenvironment. J Immunother Cancer. (2020) 8:e000189. doi: 10.1136/jitc-2019-000189, PMID: 32581043 PMC7312341

[96] Li N Li Y Han X Zhang J Han J Jiang X . LXR agonist inhibits inflammation through regulating MyD88 mRNA alternative splicing. Front Pharmacol. (2022) 13:973612. doi: 10.3389/fphar.2022.973612, PMID: 36313296 PMC9614042

[97] Liang H Shen X . LXR activation radiosensitizes non-small cell lung cancer by restricting myeloid-derived suppressor cells. Biochem Biophys Res Commun. (2020) 528:330–5. doi: 10.1016/j.bbrc.2020.04.137, PMID: 32448508

[98] Schwarz E Savardekar H Zelinskas S Mouse A Lapurga G Lyberger J . Trabectedin enhances the antitumor effects of IL-12 in triple-negative breast cancer. Cancer Immunol Res. (2025) 13:560–76. doi: 10.1158/2326-6066.CIR-24-0775, PMID: 39777457 PMC11962391

[99] Tanaka M Siemann DW . Gas6/Axl signaling pathway in the tumor immune microenvironment. Cancers (Basel). (2020) 12:1850. doi: 10.3390/cancers12071850, PMID: 32660000 PMC7408754

[100] Lv Y Zhu J Ge S Jiang T Xu Y Yao W . The AXL-mediated modulation of myeloid-derived suppressor cells (MDSC) in nasopharyngeal carcinoma. Med Oncol. (2024) 42:17. doi: 10.1007/s12032-024-02561-9, PMID: 39592496

[101] Falcone I Conciatori F Bazzichetto C Bria E Carbognin L Malaguti P . AXL receptor in breast cancer: Molecular involvement and therapeutic limitations. Int J Mol Sci. (2020) 21:8419. doi: 10.3390/ijms21228419, PMID: 33182542 PMC7696061

[102] Holtzhausen A Harris W Ubil E Hunter DM Zhao J Zhang Y . TAM family receptor kinase inhibition reverses MDSC-mediated suppression and augments anti-PD-1 therapy in melanoma. Cancer Immunol Res. (2019) 7:1672–86. doi: 10.1158/2326-6066.CIR-19-0008, PMID: 31451482 PMC6943983

[103] Du W Huang H Sorrelle N Brekken RA . Sitravatinib potentiates immune checkpoint blockade in refractory cancer models. JCI Insight. (2018) 3:e124184. doi: 10.1172/jci.insight.124184, PMID: 30385724 PMC6238734

[104] Ouerdani A Valenzuela B Treijtel N Haddish-Berhane N Desphande S Srinivasan S . Evaluation of bruton’s tyrosine kinase (BTK) inhibition with alternative doses of ibrutinib in subjects with chronic lymphocytic leukemia (CLL). Cancer Chemother Pharmacol. (2025) 95:38. doi: 10.1007/s00280-025-04753-0, PMID: 40019563 PMC11870975

[105] Stiff A Trikha P Wesolowski R Kendra K Hsu V Uppati S . Myeloid-derived suppressor cells express bruton’s tyrosine kinase and can be depleted in tumor-bearing hosts by ibrutinib treatment. Cancer Res. (2016) 76:2125–36. doi: 10.1158/0008-5472.CAN-15-1490, PMID: 26880800 PMC4873459

[106] Ferrer G Jung B Chiu PY Aslam R Palacios F Mazzarello AN . Myeloid-derived suppressor cell subtypes differentially influence T-cell function, T-helper subset differentiation, and clinical course in CLL. Leukemia. (2021) 35:3163–75. doi: 10.1038/s41375-021-01249-7, PMID: 33935280 PMC8550941

[107] Overman M Javle M Davis RE Vats P Kumar-Sinha C Xiao L . Randomized phase II study of the Bruton tyrosine kinase inhibitor acalabrutinib, alone or with pembrolizumab in patients with advanced pancreatic cancer. J Immunother Cancer. (2020) 8:e000587. doi: 10.1136/jitc-2020-000587, PMID: 32114502 PMC7057435

[108] Sun SH Angell CD Savardekar H Sundi D Abood D Benner B . BTK inhibition potentiates anti-PD-L1 treatment in murine melanoma: Potential role for MDSC modulation in immunotherapy. Cancer Immunol Immunother. (2023) 72:3461–74. doi: 10.1007/s00262-023-03497-1, PMID: 37528320 PMC10592087

[109] Solman IG Blum LK Burger JA Kipps TJ Dean JP James DF . Impact of long-term ibrutinib treatment on circulating immune cells in previously untreated chronic lymphocytic leukemia. Leuk Res. (2021) 102:106520. doi: 10.1016/j.leukres.2021.106520, PMID: 33611131

[110] Schwarz E Benner B Wesolowski R Quiroga D Good L Sun SH . Inhibition of bruton’s tyrosine kinase with PD-1 blockade modulates T cell activation in solid tumors. JCI Insight. (2024) 9:e169927. doi: 10.1172/jci.insight.169927, PMID: 39513363 PMC11601564

[111] Chang YC Chiang YH Hsu K Chuang CK Kao CW Chang YF . Activated naïve γδ T cells accelerate deep molecular response to BCR-ABL inhibitors in patients with chronic myeloid leukemia. Blood Cancer J. (2021) 11:182. doi: 10.1038/s41408-021-00572-7, PMID: 34785653 PMC8595379

[112] Giallongo C Parrinello NL La Cava P Camiolo G Romano A Scalia M . Monocytic myeloid-derived suppressor cells as prognostic factor in chronic myeloid leukaemia patients treated with dasatinib. J Cell Mol Med. (2018) 22:1070–80. doi: 10.1111/jcmm.13326, PMID: 29218828 PMC5783858

[113] Alves R McArdle SEB Vadakekolathu J Gonçalves AC Freitas-Tavares P Pereira A . Flow cytometry and targeted immune transcriptomics identify distinct profiles in patients with chronic myeloid leukemia receiving tyrosine kinase inhibitors with or without interferon-α. J Transl Med. (2020) 18:2. doi: 10.1186/s12967-019-02194-x, PMID: 31900171 PMC6941328

[114] Koller P Baran N Harutyunyan K Cavazos A Mallampati S Chin RL . PD-1 blockade in combination with dasatinib potentiates induction of anti-acute lymphocytic leukemia immunity. J Immunother Cancer. (2023) 11:e006619. doi: 10.1136/jitc-2022-006619, PMID: 37793852 PMC10551962

[115] Khaki Bakhtiarvand V Ramezani-Ali Akbari K Amir Jalali S Hojjat-Farsangi M Jeddi-Tehrani M Shokri F . Myeloid-derived suppressor cells (MDSCs) depletion by cabozantinib improves the efficacy of anti-HER2 antibody-based immunotherapy in a 4T1-HER2 murine breast cancer model. Int Immunopharmacol. (2022) 113:109470. doi: 10.1016/j.intimp.2022.109470, PMID: 36435059

[116] Li C Xiong L Yang Y Jiang P Wang J Li M . Sorafenib enhanced the function of myeloid-derived suppressor cells in hepatocellular carcinoma by facilitating PPARα-mediated fatty acid oxidation. Mol Cancer. (2025) 24:34. doi: 10.1186/s12943-025-02238-5, PMID: 39876004 PMC11773820

[117] Li J Yuan S Norgard RJ Yan F Sun YH Kim IK . Epigenetic and transcriptional control of the epidermal growth factor receptor regulates the tumor immune microenvironment in pancreatic cancer. Cancer Discov. (2021) 11:736–53. doi: 10.1158/2159-8290.CD-20-0519, PMID: 33158848 PMC7933070

[118] Yuan M Zhai Y Men Y Zhao M Sun X Ma Z . Anlotinib enhances the antitumor activity of high-dose irradiation combined with anti-PD-L1 by potentiating the tumor immune microenvironment in murine lung cancer. Oxid Med Cell Longev. (2022) 2022:5479491. doi: 10.1155/2022/5479491, PMID: 35154567 PMC8825674

[119] Ozbay Kurt FG Lasser S Arkhypov I Utikal J Umansky V . Enhancing immunotherapy response in melanoma: Myeloid-derived suppressor cells as a therapeutic target. J Clin Invest. (2023) 133:e170762. doi: 10.1172/JCI170762, PMID: 37395271 PMC10313369

[120] Greene S Robbins Y Mydlarz WK Huynh AP Schmitt NC Friedman J . Inhibition of MDSC trafficking with SX-682, a CXCR1/2 inhibitor, enhances NK-cell immunotherapy in Head and neck cancer models. Clin Cancer Res. (2020) 26:1420–31. doi: 10.1158/1078-0432.CCR-19-2625, PMID: 31848188 PMC7073293

[121] Patel SP Dimou A Victor AI Mooradian M Buchbinder EI Hernandez-Aya LF . Safety and efficacy of first-in-class CXCR1/2 inhibitor SX-682 in combination with pembrolizumab (pem) in patients (pts) with metastatic melanoma (mMEL) with disease progression on anti–PD-1 therapy. J Clin Oncol. (2024) 42:9508–. doi: 10.1200/JCO.2024.42.16_suppl.9508, PMID: 41529214

[122] Dunne RF Ullman NA Belt BA Ruffolo LI Burchard P Hezel AF . A phase I study to evaluate the safety and tolerability of SX-682 in combination with PD-1 inhibitor as maintenance therapy for unresectable pancreatic adenocarcinoma. J Clin Oncol. (2022) 40:TPS631–TPS: TPS631–. doi: 10.1200/JCO.2022.40.4_suppl.TPS631, PMID: 41529214

[123] Yang L Wang B Qin J Zhou H Majumdar APN Peng F . Blockade of CCR5-mediated myeloid derived suppressor cell accumulation enhances anti-PD1 efficacy in gastric cancer. Immunopharmacol Immunotoxicol. (2018) 40:91–7. doi: 10.1080/08923973.2017.1417997, PMID: 29303012

[124] Jiang K Li J Zhang J Wang L Zhang Q Ge J . SDF-1/CXCR4 axis facilitates myeloid-derived suppressor cells accumulation in osteosarcoma microenvironment and blunts the response to anti-PD-1 therapy. Int Immunopharmacol. (2019) 75:105818. doi: 10.1016/j.intimp.2019.105818, PMID: 31437795

[125] Zeng Y Li B Liang Y Reeves PM Qu X Ran C . Dual blockade of CXCL12-CXCR4 and PD-1-PD-L1 pathways prolongs survival of ovarian tumor-bearing mice by prevention of immunosuppression in the tumor microenvironment. FASEB J. (2019) 33:6596–608. doi: 10.1096/fj.201802067RR, PMID: 30802149 PMC6463916

[126] Bockorny B Semenisty V Macarulla T Borazanci E Wolpin BM Stemmer SM . BL-8040, a CXCR4 antagonist, in combination with pembrolizumab and chemotherapy for pancreatic cancer: The COMBAT trial. Nat Med. (2020) 26:878–85. doi: 10.1038/s41591-020-0880-x, PMID: 32451495

[127] Qian J Ryeom S Daugherty BL Lederman S Wang TC . 481 A CXCR4 partial agonist TFF2-MSA sensitized advanced gastric cancer to PD-1 blockade by systematically reducing PMN-MDSC accumulation, immunosuppression, and generation in bone marrow. J Immunother Cancer. (2023). 11:540–540. doi: 10.1136/jitc-2023-SITC2023.0481, PMID: 41686241

[128] Qian J Ryeom S Daugherty BL Lederman SM Wang TC . Effect of MDSC-targeted TFF2-MSA with PD-1 blockade therapy in advanced gastric cancer models. J Clin Oncol. (2023) 41:e16037–e:e16037–. doi: 10.1200/JCO.2023.41.16_suppl.e16037, PMID: 40260994

[129] Blattner C Fleming V Weber R Himmelhan B Altevogt P Gebhardt C . CCR5+ myeloid-derived suppressor cells are enriched and activated in melanoma lesions. Cancer Res. (2018) 78:157–67. doi: 10.1158/0008-5472.CAN-17-0348, PMID: 29089297

[130] Chen J Lin Q Lan R Wu J Wang Z Chen R . A CCR5 antagonist enhances the radiosensitivity of hepatocarcinoma in a mouse model. J Radiat Res. (2025) 66:396–407. doi: 10.1093/jrr/rraf035, PMID: 40650599 PMC12283518

[131] Peng Y Zhang J Zhang T Wang C Bai J Li Y . S100A4 mediates the accumulation and functions of myeloid-derived suppressor cells via GP130/JAK2/STAT3 signaling in acute myeloid leukemia. Biochim Biophys Acta Mol Basis Dis. (2025) 1871:167498. doi: 10.1016/j.bbadis.2024.167498, PMID: 39243827

[132] Zhang X Lou Y Zheng S Chang X . HCC-derived CX3CL1 affects hepatocellular carcinoma prognosis and CX3CR1 + MDSC infiltration. Eur J Med Res. (2025) 30:153. doi: 10.1186/s40001-025-02410-z, PMID: 40051011 PMC11884201

[133] Zhang Z Huang W Hu D Jiang J Zhang J Wu Z . E-twenty-six-specific sequence variant 5 (ETV5) facilitates hepatocellular carcinoma progression and metastasis through enhancing polymorphonuclear myeloid-derived suppressor cell (PMN-MDSC)-mediated immunosuppression. Gut. (2025) 74:1137–49. doi: 10.1136/gutjnl-2024-333944, PMID: 40015948

[134] Günther S Fagone P Jalce G Atanasov AG Guignabert C Nicoletti F . Role of MIF and D-DT in immune-inflammatory, autoimmune, and chronic respiratory diseases: From pathogenic factors to therapeutic targets. Drug Discov Today. (2019) 24:428–39. doi: 10.1016/j.drudis.2018.11.003, PMID: 30439447

[135] Alban TJ Bayik D Otvos B Rabljenovic A Leng L Jia-Shiun L . Glioblastoma myeloid-derived suppressor cell subsets express differential macrophage migration inhibitory factor receptor profiles that can be targeted to reduce immune suppression. Front Immunol. (2020) 11:1191. doi: 10.3389/fimmu.2020.01191, PMID: 32625208 PMC7315581

[136] Kumar R de Mooij T Peterson TE Kaptzan T Johnson AJ Daniels DJ . Modulating glioma-mediated myeloid-derived suppressor cell development with sulforaphane. PloS One. (2017) 12:e0179012. doi: 10.1371/journal.pone.0179012, PMID: 28666020 PMC5493295

[137] Yang Z Guo J Cui K Du Y Zhao H Zhu L . Thymosin alpha-1 blocks the accumulation of myeloid suppressor cells in NSCLC by inhibiting VEGF production. BioMed Pharmacother. (2020) 131:110740. doi: 10.1016/j.biopha.2020.110740, PMID: 32942159

[138] Shi F Qiu H Yan J Ke C Li Y . Effect of thymalfasin on myeloid-derived suppressor cells in patients with non-small cell lung cancer. Am J Transl Res. (2024) 16:1790–7. doi: 10.62347/QSWS7848, PMID: 38883367 PMC11170616

[139] Sadhukhan P Feng M Illingworth E Sloma I Ooki A Matoso A . YAP1 induces bladder cancer progression and promotes immune evasion through IL-6/STAT3 pathway and CXCL deregulation. J Clin Invest. (2024) 135:e171164. doi: 10.1172/JCI171164, PMID: 39630608 PMC11735109

[140] Steinberg SM Shabaneh TB Zhang P Martyanov V Li Z Malik BT . Myeloid cells that impair immunotherapy are restored in melanomas with acquired resistance to BRAF inhibitors. Cancer Res. (2017) 77:1599–610. doi: 10.1158/0008-5472.CAN-16-1755, PMID: 28202513 PMC5380540

[141] Wang L Hu D Xie B Xie L . Blockade of Myd88 signaling by a novel MyD88 inhibitor prevents colitis-associated colorectal cancer development by impairing myeloid-derived suppressor cells. Investig New Drugs. (2022) 40:506–18. doi: 10.1007/s10637-022-01218-6, PMID: 35089465 PMC9098617

[142] Tong X Xiao M Yang J Xu J Wang W Yu X . The TMBIM1-YBX1 axis orchestrates MDSC recruitment and immunosuppressive microenvironment in pancreatic cancer. Theranostics. (2025) 15:2794–813. doi: 10.7150/thno.111180, PMID: 40083936 PMC11898282

[143] Ford JW Gonzalez-Cotto M MacFarlane AW Peri S Howard OMZ Subleski JJ . Tumor-infiltrating myeloid cells co-express TREM1 and TREM2 and elevated TREM-1 associates with disease progression in renal cell carcinoma. Front Oncol. (2021) 11:662723. doi: 10.3389/fonc.2021.662723, PMID: 35223446 PMC8867210

[144] Ajith A Mamouni K Horuzsko DD Musa A Dzutsev AK Fang JR . Targeting TREM1 augments antitumor T cell immunity by inhibiting myeloid-derived suppressor cells and restraining anti-PD-1 resistance. J Clin Invest. (2023) 133:e167951. doi: 10.1172/JCI167951, PMID: 37651197 PMC10617775

[145] Wu J Qian P Han Y Xu C Xia M Zhan P . GLP1 alleviates oleic acid-propelled lipocalin-2 generation by tumor-infiltrating CD8+ T cells to reduce polymorphonuclear MDSC recruitment and enhances viral immunotherapy in pancreatic cancer. Cell Mol Immunol. (2025) 22:282–99. doi: 10.1038/s41423-025-01260-3, PMID: 39910336 PMC11868399

[146] Tobin RP Cogswell DT Cates VM Davis DM Borgers JSW Van Gulick RJ . Targeting MDSC differentiation using ATRA: A Phase I/II clinical trial combining pembrolizumab and all-trans retinoic acid for metastatic melanoma. Clin Cancer Res. (2023) 29:1209–19. doi: 10.1158/1078-0432.CCR-22-2495, PMID: 36378549 PMC10073240

[147] Liang Y Wang W Zhu X Yu M Zhou C . Inhibition of myeloid-derived suppressive cell function with all-trans retinoic acid enhanced anti-PD-L1 efficacy in cervical cancer. Sci Rep. (2022) 12:9619. doi: 10.1038/s41598-022-13855-1, PMID: 35688951 PMC9187659

[148] Tobin RP Jordan KR Robinson WA Davis D Borges VF Gonzalez R . Targeting myeloid-derived suppressor cells using all-trans retinoic acid in melanoma patients treated with ipilimumab. Int Immunopharmacol. (2018) 63:282–91. doi: 10.1016/j.intimp.2018.08.007, PMID: 30121453 PMC6134177

[149] Zheng A Xie F Shi S Liu S Long J Xu Y . Sustained drug release from liposomes for the remodeling of systemic immune homeostasis and the tumor microenvironment. Front Immunol. (2022) 13:829391. doi: 10.3389/fimmu.2022.829391, PMID: 35493504 PMC9039229

[150] Huang R Zheng A Xu Y Wu J . 1002P A phase Ia study of the myeloid-derived suppressor cell modulator HF1K16 in refractory and metastatic cancer patients: Preliminary efficacy and safety. Ann Oncol. (2024) 35:S682. doi: 10.1016/j.annonc.2024.08.1061, PMID: 41732981

[151] Fleet JC Burcham GN Calvert RD Elzey BD Ratliff TL . 1α, 25 Dihydroxyvitamin D (1,25(OH)2D) inhibits the T cell suppressive function of myeloid derived suppressor cells (MDSC). J Steroid Biochem Mol Biol. (2020) 198:105557. doi: 10.1016/j.jsbmb.2019.105557, PMID: 31783150 PMC8041088

[152] Chomczyk M Gazzola L Dash S Firmanty P George BS Mohanty V . Impact of p53-associated acute myeloid leukemia hallmarks on metabolism and the immune environment. Front Pharmacol. (2024) 15:1409210. doi: 10.3389/fphar.2024.1409210, PMID: 39161899 PMC11330794

[153] Sharma MD Rodriguez PC Koehn BH Baban B Cui Y Guo G . Activation of p53 in immature myeloid precursor cells controls differentiation into ly6c+cd103+ monocytic antigen-presenting cells in tumors. Immunity. (2018) 48:91–106.e6. doi: 10.1016/j.immuni.2017.12.014, PMID: 29343444 PMC6005382

[154] Deng Y Yang J Qian J Liu R Huang E Wang Y . TLR1/TLR2 signaling blocks the suppression of monocytic myeloid-derived suppressor cell by promoting its differentiation into M1-type macrophage. Mol Immunol. (2019) 112:266–73. doi: 10.1016/j.molimm.2019.06.006, PMID: 31212097

[155] Li S Li F Xu L Liu X Zhu X Gao W . TLR2 agonist promotes myeloid-derived suppressor cell polarization via Runx1 in hepatocellular carcinoma. Int Immunopharmacol. (2022) 111:109168. doi: 10.1016/j.intimp.2022.109168, PMID: 35998504

[156] Liu Z Xie Y Xiong Y Liu S Qiu C Zhu Z . TLR 7/8 agonist reverses oxaliplatin resistance in colorectal cancer via directing the myeloid-derived suppressor cells to tumoricidal M1-macrophages. Cancer Lett. (2020) 469:173–85. doi: 10.1016/j.canlet.2019.10.020, PMID: 31629935

[157] Alsaafeen BH Ali BR Elkord E . Resistance mechanisms to immune checkpoint inhibitors: Updated insights. Mol Cancer. (2025) 24:20. doi: 10.1186/s12943-024-02212-7, PMID: 39815294 PMC11734352

[158] Davis RJ Moore EC Clavijo PE Friedman J Cash H Chen Z . Anti-PD-L1 efficacy can be enhanced by inhibition of myeloid-derived suppressor cells with a selective inhibitor of PI3Kδ/γ. Cancer Res. (2017) 77:2607–19. doi: 10.1158/0008-5472.CAN-16-2534, PMID: 28364000 PMC5466078

[159] Inoue H Hamasaki T Inoue K Nakao A Ebi N Minomo H . Comprehensive immunophenotyping reveals distinct tumor microenvironment alterations in anti-PD-1 sensitive and resistant syngeneic mouse model. Sci Rep. (2025) 15:8311. doi: 10.1038/s41598-025-91979-w, PMID: 40064915 PMC11894063

[160] Jeong H Koh J Kim S Yim J Song SG Kim H . Cell-intrinsic PD-L1 signaling drives immunosuppression by myeloid-derived suppressor cells through IL-6/Jak/Stat3 in PD-L1-high lung cancer. J Immunother Cancer. (2025) 13:e010612. doi: 10.1136/jitc-2024-010612, PMID: 40050048 PMC11887297

[161] Qin G Liu S Liu J Hu H Yang L Zhao Q . Overcoming resistance to immunotherapy by targeting GPR84 in myeloid-derived suppressor cells. Signal Transduct Target Ther. (2023) 8:164. doi: 10.1038/s41392-023-01388-6, PMID: 37105980 PMC10140025

[162] Zhang M Wang L Liu W Wang T De Sanctis F Zhu L . Targeting inhibition of accumulation and function of myeloid-derived suppressor cells by artemisinin via PI3K/AKT, mTOR, and MAPK pathways enhances anti-PD-L1 immunotherapy in melanoma and liver tumors. J Immunol Res. (2022) 2022:2253436. doi: 10.1155/2022/2253436, PMID: 35785030 PMC9247850

[163] Tanaka K Chamoto K Saeki S Hatae R Ikematsu Y Sakai K . Combination bezafibrate and nivolumab treatment of patients with advanced non–small cell lung cancer. Sci Transl Med. (2022) 14:eabq0021. doi: 10.1126/scitranslmed.abq0021, PMID: 36516270

[164] Wan H Xu B Zhu N Ren B . PGC-1α activator-induced fatty acid oxidation in tumor-infiltrating CTLs enhances effects of PD-1 blockade therapy in lung cancer. Tumori. (2020) 106:55–63. doi: 10.1177/0300891619868287, PMID: 31451071

[165] Pan J Li J Zhang Q Huang M Wang Y You M . Bezafibrate-driven mitochondrial targeting enhances antitumor immunity and prevents lung cancer via CD8+ T cell infiltration and MDSC reduction. Front Immunol. (2025) 16:1539808. doi: 10.3389/fimmu.2025.1539808, PMID: 40303399 PMC12037589

[166] Mita MM Mita AC Chmielowski B Hamilton EP Pant S Waltzman RJ . Pharmacodynamic and clinical activity of RGX-104, a first-in-class immunotherapy targeting the liver-X nuclear hormone receptor (LXR), in patients with refractory Malignancies. J Clin Oncol. (2018) 36:3095–. doi: 10.1200/JCO.2018.36.15_suppl.3095, PMID: 33204107

[167] First-in-human Phase I study to evaluate safety, tolerability and antineoplastic activity of OATD-02. In: Patients with selected advanced and/or metastatic solid tumours. Warsaw, Poland: Molecure S.A. Available online at: https://clinicaltrials.gov/study/NCT05759923 (Accessed February 24, 2025).

[168] Naing A Papadopoulos KP Pishvaian MJ Rahma O Hanna GJ Garralda E . First-in-human phase 1 study of the arginase inhibitor INCB001158 alone or combined with pembrolizumab in patients with advanced or metastatic solid tumours. BMJ Oncol. (2024) 3:e000249. doi: 10.1136/bmjonc-2023-000249, PMID: 39886141 PMC11235002

[169] Weed DT Zilio S Reis IM Sargi Z Abouyared M Gomez-Fernandez CR . The reversal of immune exclusion mediated by tadalafil and an anti-tumor vaccine also induces PDL1 upregulation in recurrent Head and neck squamous cell carcinoma: Interim analysis of a Phase I clinical trial. Front Immunol. (2019) 10:1206. doi: 10.3389/fimmu.2019.01206, PMID: 31214178 PMC6554471

[170] Benguigui M Vorontsova A Timaner M Levin S Haj-Shomaly J Deo A . Bv8 blockade sensitizes anti-PD1 therapy resistant tumors. Front Immunol. (2022) 13:903591. doi: 10.3389/fimmu.2022.903591, PMID: 35874722 PMC9301046

[171] Nguyen NT Mitsuhashi A Ogino H Kozai H Yoneda H Afroj T . S-1 eliminates MDSCs and enhances the efficacy of PD-1 blockade via regulation of tumor-derived Bv8 and S100A8 in thoracic tumor. Cancer Sci. (2023) 114:384–98. doi: 10.1111/cas.15620, PMID: 36285504 PMC9899614

[172] Zhang Y Hu J Ji K Jiang S Dong Y Sun L . CD39 inhibition and VISTA blockade may overcome radiotherapy resistance by targeting exhausted CD8+ T cells and immunosuppressive myeloid cells. Cell Rep Med. (2023) 4:101151. doi: 10.1016/j.xcrm.2023.101151, PMID: 37567173 PMC10439278

[173] Marguier A Laheurte C Lecoester B Malfroy M Boullerot L Renaudin A . TIE-2 signaling activation by angiopoietin 2 on myeloid-derived suppressor cells promotes melanoma-specific T-cell inhibition. Front Immunol. (2022) 13:932298. doi: 10.3389/fimmu.2022.932298, PMID: 35935946 PMC9353943

[174] Verzoni E Rodolfo M Vallacchi V Todoerti K Zucali PA Perrucci B . Association of myeloid-derived suppressor cell (MDSC) dynamics with clinical response to nivolumab in metastatic clear cell renal carcinoma patients (mRCC): Results from the I-RENE Meet-URO 8 study. J Clin Oncol. (2025) 43:586–. doi: 10.1200/JCO.2025.43.5_suppl.586

[175] Long AH Highfill SL Cui Y Smith JP Walker AJ Ramakrishna S . Reduction of MDSCs with all-trans retinoic acid improves CAR therapy efficacy for sarcomas. Cancer Immunol Res. (2016) 4:869–80. doi: 10.1158/2326-6066.CIR-15-0230, PMID: 27549124 PMC5050151

[176] Tumino N Weber G Besi F Del Bufalo F Bertaina V Paci P . Polymorphonuclear myeloid-derived suppressor cells impair the anti-tumor efficacy of GD2.CAR T-cells in patients with neuroblastoma. J Hematol Oncol. (2021) 14:191. doi: 10.1186/s13045-021-01193-0, PMID: 34772439 PMC8588686

[177] Frost BF Shestova O Shen F Sharpe C Byrd JC Gill SI . Abstract 57: Ibrutinib improves chimeric antigen receptor T cell control of leukemia by inhibiting myeloid-derived suppressor cells. Cancer Res. (2024) 84:57–. Abstract 57. doi: 10.1158/1538-7445.AM2024-57, PMID: 41680580

[178] Sun R Luo H Su J Di S Zhou M Shi B . Olaparib suppresses MDSC recruitment via SDF1α/CXCR4 axis to improve the anti-tumor efficacy of CAR-T cells on breast cancer in mice. Mol Ther. (2021) 29:60–74. doi: 10.1016/j.ymthe.2020.09.034, PMID: 33010818 PMC7791086

[179] Sun R Sun Y Wu C Liu Y Zhou M Dong Y . CXCR4-modified CAR-T cells suppresses MDSCs recruitment via STAT3/NF-κB/SDF-1α axis to enhance efficacy against pancreatic cancer. Mol Ther. (2023) 31:3193–209. doi: 10.1016/j.ymthe.2023.09.010, PMID: 37735875 PMC10638076

[180] Lu M Zhang X Gao X Sun S Wei X Hu X . Lenvatinib enhances T cell immunity and the efficacy of adoptive chimeric antigen receptor-modified T cells by decreasing myeloid-derived suppressor cells in cancer. Pharmacol Res. (2021) 174:105829. doi: 10.1016/j.phrs.2021.105829, PMID: 34411731

[181] Di S Zhou M Pan Z Sun R Chen M Jiang H . Combined adjuvant of poly I:C improves antitumor effects of CAR-T cells. Front Oncol. (2019) 9:241. doi: 10.3389/fonc.2019.00241, PMID: 31058074 PMC6481273

[182] Azeez SS Yashooa RK Smail SW Salihi A Ali AS Mamand S . Advancing CAR-based cell therapies for solid tumours: Challenges, therapeutic strategies, and perspectives. Mol Cancer. (2025) 24:191. doi: 10.1186/s12943-025-02386-8, PMID: 40624498 PMC12232864

[183] Nalawade SA Shafer P Bajgain P McKenna MK Ali A Kelly L . Selectively targeting myeloid-derived suppressor cells through TRAIL receptor 2 to enhance the efficacy of CAR T cell therapy for treatment of breast cancer. J Immunother Cancer. (2021) 9:e003237. doi: 10.1136/jitc-2021-003237, PMID: 34815355 PMC8611441

[184] Glabman RA Olkowski CP Minor HA Bassel LL Kedei N Choyke PL . Tumor suppression by anti-fibroblast activation protein near-infrared photoimmunotherapy targeting cancer-associated fibroblasts. Cancers. (2024) 16:449. doi: 10.3390/cancers16020449, PMID: 38275890 PMC10813865

[185] Liu Y Sun Y Wang P Li S Dong Y Zhou M . FAP-targeted CAR-T suppresses MDSCs recruitment to improve the antitumor efficacy of claudin18.2-targeted CAR-T against pancreatic cancer. J Transl Med. (2023) 21:255. doi: 10.1186/s12967-023-04080-z, PMID: 37046312 PMC10091631

[186] Parihar R Rivas C Huynh M Omer B Lapteva N Metelitsa LS . NK cells expressing a chimeric activating receptor eliminate MDSCs and rescue impaired CAR-T cell activity against solid tumors. Cancer Immunol Res. (2019) 7:363–75. doi: 10.1158/2326-6066.CIR-18-0572, PMID: 30651290 PMC7906796

[187] Bleve A Consonni FM Porta C Garlatti V Sica A . Evolution and targeting of myeloid suppressor cells in cancer: A translational perspective. Cancers (Basel). (2022) 14:510. doi: 10.3390/cancers14030510, PMID: 35158779 PMC8833347

[188] Cheng CH Shi SS . Artificial intelligence in cancer: applications, challenges, and future perspectives. Mol Cancer. (2025) 24:274. doi: 10.1186/s12943-025-02450-3, PMID: 41168799 PMC12574039

[189] Suarez-Carmona M Halama N . Neoadjuvant combination immunotherapy in MSI/dMMR colorectal cancer. Trends Cancer. (2024) 10:1093–4. doi: 10.1016/j.trecan.2024.10.006, PMID: 39448335

[190] Dominguez GA Roop J Polo A Campisi A Gabrilovich DI Kumar A . Combining the immunophenotyping of MDSCs and lymphocytes with artificial intelligence (AI) to predict early-stage breast cancer. Cancer Immunol Res. (2020) 8:A12. doi: 10.1158/2326-6074.TUMIMM18-A12, PMID: 41680580

[191] Ren S Lu Y Zhang G Xie K Chen D Cai X . Integration of graph neural networks and multi-omics analysis identify the predictive factor and key gene for immunotherapy response and prognosis of bladder cancer. J Transl Med. (2024) 22:1141. doi: 10.1186/s12967-024-05976-0, PMID: 39716185 PMC11664855

[192] Acs B Ahmed FS Gupta S . An open source automated tumor infiltrating lymphocyte algorithm for prognosis in melanoma. Nat Commun. (2019) 10:5440. doi: 10.1038/s41467-019-13043-2, PMID: 31784511 PMC6884485

[193] Furudate K Takahashi K . LLM-scCurator: data-centric feature distillation for zero-shot cell-type annotation. bioRxiv. (2025) doi: 10.64898/2025.12.28.696778, PMID: 41453657

[194] Yu Y Cai G Lin R Wang Z Chen Y Tan Y . Multimodal data fusion AI model uncovers tumor microenvironment immunotyping heterogeneity and enhanced risk stratification of breast cancer. MedComm(2020). (2024) 5:e70023. doi: 10.1002/mco2.70023, PMID: 39669975 PMC11635117

[195] Kailayangiri S Altvater B Wiebel M Jamitzky S Rossig C . Overcoming heterogeneity of antigen expression for effective CAR T cell targeting of cancers. Cancers (Basel). (2020) 12:1075. doi: 10.3390/cancers12051075, PMID: 32357417 PMC7281243

[196] Möller M Orth V Umansky V Hetjens S Braun V Reißfelder C . Myeloid-derived suppressor cells in peripheral blood as predictive biomarkers in patients with solid tumors undergoing immune checkpoint therapy: Systematic review and meta-analysis. Front Immunol. (2024) 15:1403771. doi: 10.3389/fimmu.2024.1403771, PMID: 38855104 PMC11157008

[197] Gyau BB Wang J Wu W Scull B Major AM Jin W . Multiplex imaging mass cytometry reveals prognostic immunosuppressive subpopulations and macrophage-driven metastasis in osteosarcoma. Cancers. (2025) 17:2780. doi: 10.3390/cancers17172780, PMID: 40940877 PMC12427482

[198] Xu H Li S Liu Y Sung YY Zhou Y Wu H . A novel pH-sensitive nanoparticles encapsulating anti-PD-1 antibody and MDK-siRNA overcome immune checkpoint blockade resistance in HCC via reshaping immunosuppressive TME. J Exp Clin Cancer Res. (2025) 44:148. doi: 10.1186/s13046-025-03396-6, PMID: 40380202 PMC12082952

[199] Elter A Yanakieva D Fiebig D Hallstein K Becker S Betz U . Protease-activation of Fc-masked therapeutic antibodies to alleviate off-tumor cytotoxicity. Front Immunol. (2021) 12:715719. doi: 10.3389/fimmu.2021.715719, PMID: 34413859 PMC8369199

[200] Guo C Lin L Wang Y Jing J Gong Q Luo K . Nano drug delivery systems for advanced immune checkpoint blockade therapy. Theranostics. (2025) 15:5440–80. doi: 10.7150/thno.112475, PMID: 40303342 PMC12036873

[201] Yutao C Desheng C Zhixing L Haoyuan Y Haobin S Yongwei H . Immune checkpoint inhibitors in hepatocellular carcinoma therapy: Resistance mechanisms, liver transplantation challenges and management strategies. Cancer Drug Resistance (2025) 8:48. doi: 10.20517/cdr.2025.120, PMID: 41019983 PMC12462397

[202] Cheema PK Iafolla MAJ Abdel-Qadir H Bellini AB Chatur N Chandok N . Managing select immune-related adverse events in patients treated with immune checkpoint inhibitors. Curr Oncol. (2024) 31:6356–83. doi: 10.3390/curroncol31100473, PMID: 39451777 PMC11506662

